# Measurement of the ${\mathrm {t}\overline{\mathrm {t}}}$ production cross section, the top quark mass, and the strong coupling constant using dilepton events in pp collisions at $\sqrt{s}=13\,\text {Te}\text {V}$

**DOI:** 10.1140/epjc/s10052-019-6863-8

**Published:** 2019-04-29

**Authors:** A. M. Sirunyan, A. Tumasyan, W. Adam, F. Ambrogi, E. Asilar, T. Bergauer, J. Brandstetter, M. Dragicevic, J. Erö, A. Escalante Del Valle, M. Flechl, R. Frühwirth, V. M. Ghete, J. Hrubec, M. Jeitler, N. Krammer, I. Krätschmer, D. Liko, T. Madlener, I. Mikulec, N. Rad, H. Rohringer, J. Schieck, R. Schöfbeck, M. Spanring, D. Spitzbart, W. Waltenberger, J. Wittmann, C.-E. Wulz, M. Zarucki, V. Chekhovsky, V. Mossolov, J. Suarez Gonzalez, E. A. De Wolf, D. Di Croce, X. Janssen, J. Lauwers, A. Lelek, M. Pieters, H. Van Haevermaet, P. Van Mechelen, N. Van Remortel, S. Abu Zeid, F. Blekman, J. D’Hondt, J. De Clercq, K. Deroover, G. Flouris, D. Lontkovskyi, S. Lowette, I. Marchesini, S. Moortgat, L. Moreels, Q. Python, K. Skovpen, S. Tavernier, W. Van Doninck, P. Van Mulders, I. Van Parijs, D. Beghin, B. Bilin, H. Brun, B. Clerbaux, G. De Lentdecker, H. Delannoy, B. Dorney, G. Fasanella, L. Favart, A. Grebenyuk, A. K. Kalsi, T. Lenzi, J. Luetic, N. Postiau, E. Starling, L. Thomas, C. Vander Velde, P. Vanlaer, D. Vannerom, Q. Wang, T. Cornelis, D. Dobur, A. Fagot, M. Gul, I. Khvastunov, D. Poyraz, C. Roskas, D. Trocino, M. Tytgat, W. Verbeke, B. Vermassen, M. Vit, N. Zaganidis, H. Bakhshiansohi, O. Bondu, G. Bruno, C. Caputo, P. David, C. Delaere, M. Delcourt, A. Giammanco, G. Krintiras, V. Lemaitre, A. Magitteri, K. Piotrzkowski, A. Saggio, M. Vidal Marono, P. Vischia, J. Zobec, F. L. Alves, G. A. Alves, G. Correia Silva, C. Hensel, A. Moraes, M. E. Pol, P. Rebello Teles, E. Belchior Batista Das Chagas, W. Carvalho, J. Chinellato, E. Coelho, E. M. Da Costa, G. G. Da Silveira, D. De Jesus Damiao, C. De Oliveira Martins, S. Fonseca De Souza, H. Malbouisson, D. Matos Figueiredo, M. Melo De Almeida, C. Mora Herrera, L. Mundim, H. Nogima, W. L. Prado Da Silva, L. J. Sanchez Rosas, A. Santoro, A. Sznajder, M. Thiel, E. J. Tonelli Manganote, F. Torres Da Silva De Araujo, A. Vilela Pereira, S. Ahuja, C. A. Bernardes, L. Calligaris, T. R. Fernandez Perez Tomei, E. M. Gregores, P. G. Mercadante, S. F. Novaes, SandraS. Padula, A. Aleksandrov, R. Hadjiiska, P. Iaydjiev, A. Marinov, M. Misheva, M. Rodozov, M. Shopova, G. Sultanov, A. Dimitrov, L. Litov, B. Pavlov, P. Petkov, W. Fang, X. Gao, L. Yuan, M. Ahmad, J. G. Bian, G. M. Chen, H. S. Chen, M. Chen, Y. Chen, C. H. Jiang, D. Leggat, H. Liao, Z. Liu, S. M. Shaheen, A. Spiezia, J. Tao, E. Yazgan, H. Zhang, S. Zhang, J. Zhao, Y. Ban, G. Chen, A. Levin, J. Li, L. Li, Q. Li, Y. Mao, S. J. Qian, D. Wang, Y. Wang, C. Avila, A. Cabrera, C. A. Carrillo Montoya, L. F. Chaparro Sierra, C. Florez, C. F. González Hernández, M. A. Segura Delgado, B. Courbon, N. Godinovic, D. Lelas, I. Puljak, T. Sculac, Z. Antunovic, M. Kovac, V. Brigljevic, D. Ferencek, K. Kadija, B. Mesic, M. Roguljic, A. Starodumov, T. Susa, M. W. Ather, A. Attikis, M. Kolosova, G. Mavromanolakis, J. Mousa, C. Nicolaou, F. Ptochos, P. A. Razis, H. Rykaczewski, M. Finger, M. Finger, E. Ayala, E. Carrera Jarrin, H. Abdalla, A. Mohamed, E. Salama, S. Bhowmik, A. Carvalho Antunes De Oliveira, R. K. Dewanjee, K. Ehataht, M. Kadastik, M. Raidal, C. Veelken, P. Eerola, H. Kirschenmann, J. Pekkanen, M. Voutilainen, J. Havukainen, J. K. Heikkilä, T. Järvinen, V. Karimäki, R. Kinnunen, T. Lampén, K. Lassila-Perini, S. Laurila, S. Lehti, T. Lindén, P. Luukka, T. Mäenpää, H. Siikonen, E. Tuominen, J. Tuominiemi, T. Tuuva, M. Besancon, F. Couderc, M. Dejardin, D. Denegri, J. L. Faure, F. Ferri, S. Ganjour, A. Givernaud, P. Gras, G. Hamel de Monchenault, P. Jarry, C. Leloup, E. Locci, J. Malcles, G. Negro, J. Rander, A. Rosowsky, M. Ö. Sahin, M. Titov, A. Abdulsalam, C. Amendola, I. Antropov, F. Beaudette, P. Busson, C. Charlot, R. Granier de Cassagnac, I. Kucher, A. Lobanov, J. Martin Blanco, C. Martin Perez, M. Nguyen, C. Ochando, G. Ortona, P. Paganini, J. Rembser, R. Salerno, J. B. Sauvan, Y. Sirois, A. G. Stahl Leiton, A. Zabi, A. Zghiche, J.-L. Agram, J. Andrea, D. Bloch, G. Bourgatte, J.-M. Brom, E. C. Chabert, V. Cherepanov, C. Collard, E. Conte, J.-C. Fontaine, D. Gelé, U. Goerlach, M. Jansová, A.-C. Le Bihan, N. Tonon, P. Van Hove, S. Gadrat, S. Beauceron, C. Bernet, G. Boudoul, N. Chanon, R. Chierici, D. Contardo, P. Depasse, H. El Mamouni, J. Fay, L. Finco, S. Gascon, M. Gouzevitch, G. Grenier, B. Ille, F. Lagarde, I. B. Laktineh, H. Lattaud, M. Lethuillier, L. Mirabito, S. Perries, A. Popov, V. Sordini, G. Touquet, M. Vander Donckt, S. Viret, A. Khvedelidze, Z. Tsamalaidze, C. Autermann, L. Feld, M. K. Kiesel, K. Klein, M. Lipinski, M. Preuten, M. P. Rauch, C. Schomakers, J. Schulz, M. Teroerde, B. Wittmer, A. Albert, M. Erdmann, S. Erdweg, T. Esch, R. Fischer, S. Ghosh, T. Hebbeker, C. Heidemann, K. Hoepfner, H. Keller, L. Mastrolorenzo, M. Merschmeyer, A. Meyer, P. Millet, S. Mukherjee, T. Pook, A. Pozdnyakov, M. Radziej, H. Reithler, M. Rieger, A. Schmidt, D. Teyssier, S. Thüer, G. Flügge, O. Hlushchenko, T. Kress, T. Müller, A. Nehrkorn, A. Nowack, C. Pistone, O. Pooth, D. Roy, H. Sert, A. Stahl, M. Aldaya Martin, T. Arndt, C. Asawatangtrakuldee, I. Babounikau, K. Beernaert, O. Behnke, U. Behrens, A. Bermúdez Martínez, D. Bertsche, A. A. Bin Anuar, K. Borras, V. Botta, A. Campbell, P. Connor, C. Contreras-Campana, V. Danilov, A. De Wit, M. M. Defranchis, C. Diez Pardos, D. Domínguez Damiani, G. Eckerlin, T. Eichhorn, A. Elwood, E. Eren, E. Gallo, A. Geiser, J. M. Grados Luyando, A. Grohsjean, M. Guthoff, M. Haranko, A. Harb, H. Jung, M. Kasemann, J. Keaveney, C. Kleinwort, J. Knolle, D. Krücker, W. Lange, T. Lenz, J. Leonard, K. Lipka, W. Lohmann, R. Mankel, I.-A. Melzer-Pellmann, A. B. Meyer, M. Meyer, M. Missiroli, G. Mittag, J. Mnich, V. Myronenko, S. K. Pflitsch, D. Pitzl, A. Raspereza, A. Saibel, M. Savitskyi, P. Saxena, P. Schütze, C. Schwanenberger, R. Shevchenko, A. Singh, H. Tholen, O. Turkot, A. Vagnerini, M. Van De Klundert, G. P. Van Onsem, R. Walsh, Y. Wen, K. Wichmann, C. Wissing, O. Zenaiev, R. Aggleton, S. Bein, L. Benato, A. Benecke, T. Dreyer, A. Ebrahimi, E. Garutti, D. Gonzalez, P. Gunnellini, J. Haller, A. Hinzmann, A. Karavdina, G. Kasieczka, R. Klanner, R. Kogler, N. Kovalchuk, S. Kurz, V. Kutzner, J. Lange, D. Marconi, J. Multhaup, M. Niedziela, C. E. N. Niemeyer, D. Nowatschin, A. Perieanu, A. Reimers, O. Rieger, C. Scharf, P. Schleper, S. Schumann, J. Schwandt, J. Sonneveld, H. Stadie, G. Steinbrück, F. M. Stober, M. Stöver, B. Vormwald, I. Zoi, M. Akbiyik, C. Barth, M. Baselga, S. Baur, E. Butz, R. Caspart, T. Chwalek, F. Colombo, W. De Boer, A. Dierlamm, K. El Morabit, N. Faltermann, B. Freund, M. Giffels, M. A. Harrendorf, F. Hartmann, S. M. Heindl, U. Husemann, I. Katkov, S. Kudella, S. Mitra, M. U. Mozer, Th. Müller, M. Musich, M. Plagge, G. Quast, K. Rabbertz, M. Schröder, I. Shvetsov, H. J. Simonis, R. Ulrich, S. Wayand, M. Weber, T. Weiler, C. Wöhrmann, R. Wolf, G. Anagnostou, G. Daskalakis, T. Geralis, A. Kyriakis, D. Loukas, G. Paspalaki, A. Agapitos, G. Karathanasis, P. Kontaxakis, A. Panagiotou, I. Papavergou, N. Saoulidou, K. Vellidis, K. Kousouris, I. Papakrivopoulos, G. Tsipolitis, I. Evangelou, C. Foudas, P. Gianneios, P. Katsoulis, P. Kokkas, S. Mallios, N. Manthos, I. Papadopoulos, E. Paradas, J. Strologas, F. A. Triantis, D. Tsitsonis, M. Bartók, M. Csanad, N. Filipovic, P. Major, M. I. Nagy, G. Pasztor, O. Surányi, G. I. Veres, G. Bencze, C. Hajdu, D. Horvath, Á. Hunyadi, F. Sikler, T. Á. Vámi, V. Veszpremi, G. Vesztergombi, N. Beni, S. Czellar, J. Karancsi, A. Makovec, J. Molnar, Z. Szillasi, P. Raics, Z. L. Trocsanyi, B. Ujvari, S. Choudhury, J. R. Komaragiri, P. C. Tiwari, S. Bahinipati, C. Kar, P. Mal, K. Mandal, A. Nayak, S. Roy Chowdhury, D. K. Sahoo, S. K. Swain, S. Bansal, S. B. Beri, V. Bhatnagar, S. Chauhan, R. Chawla, N. Dhingra, R. Gupta, A. Kaur, M. Kaur, S. Kaur, P. Kumari, M. Lohan, M. Meena, A. Mehta, K. Sandeep, S. Sharma, J. B. Singh, A. K. Virdi, G. Walia, A. Bhardwaj, B. C. Choudhary, R. B. Garg, M. Gola, S. Keshri, Ashok Kumar, S. Malhotra, M. Naimuddin, P. Priyanka, K. Ranjan, Aashaq Shah, R. Sharma, R. Bhardwaj, M. Bharti, R. Bhattacharya, S. Bhattacharya, U. Bhawandeep, D. Bhowmik, S. Dey, S. Dutt, S. Dutta, S. Ghosh, M. Maity, K. Mondal, S. Nandan, A. Purohit, P. K. Rout, A. Roy, G. Saha, S. Sarkar, T. Sarkar, M. Sharan, B. Singh, S. Thakur, P. K. Behera, A. Muhammad, R. Chudasama, D. Dutta, V. Jha, V. Kumar, D. K. Mishra, P. K. Netrakanti, L. M. Pant, P. Shukla, P. Suggisetti, T. Aziz, M. A. Bhat, S. Dugad, G. B. Mohanty, N. Sur, RavindraKumar Verma, S. Banerjee, S. Bhattacharya, S. Chatterjee, P. Das, M. Guchait, Sa. Jain, S. Karmakar, S. Kumar, G. Majumder, K. Mazumdar, N. Sahoo, S. Chauhan, S. Dube, V. Hegde, A. Kapoor, K. Kothekar, S. Pandey, A. Rane, A. Rastogi, S. Sharma, S. Chenarani, E. Eskandari Tadavani, S. M. Etesami, M. Khakzad, M. Mohammadi Najafabadi, M. Naseri, F. Rezaei Hosseinabadi, B. Safarzadeh, M. Zeinali, M. Felcini, M. Grunewald, M. Abbrescia, C. Calabria, A. Colaleo, D. Creanza, L. Cristella, N. De Filippis, M. De Palma, A. Di Florio, F. Errico, L. Fiore, A. Gelmi, G. Iaselli, M. Ince, S. Lezki, G. Maggi, M. Maggi, G. Miniello, S. My, S. Nuzzo, A. Pompili, G. Pugliese, R. Radogna, A. Ranieri, G. Selvaggi, A. Sharma, L. Silvestris, R. Venditti, P. Verwilligen, G. Abbiendi, C. Battilana, D. Bonacorsi, L. Borgonovi, S. Braibant-Giacomelli, R. Campanini, P. Capiluppi, A. Castro, F. R. Cavallo, S. S. Chhibra, G. Codispoti, M. Cuffiani, G. M. Dallavalle, F. Fabbri, A. Fanfani, E. Fontanesi, P. Giacomelli, C. Grandi, L. Guiducci, F. Iemmi, S. Lo Meo, S. Marcellini, G. Masetti, A. Montanari, F. L. Navarria, A. Perrotta, F. Primavera, A. M. Rossi, T. Rovelli, G. P. Siroli, N. Tosi, S. Albergo, A. Di Mattia, R. Potenza, A. Tricomi, C. Tuve, G. Barbagli, K. Chatterjee, V. Ciulli, C. Civinini, R. D’Alessandro, E. Focardi, G. Latino, P. Lenzi, M. Meschini, S. Paoletti, L. Russo, G. Sguazzoni, D. Strom, L. Viliani, L. Benussi, S. Bianco, F. Fabbri, D. Piccolo, F. Ferro, R. Mulargia, E. Robutti, S. Tosi, A. Benaglia, A. Beschi, F. Brivio, V. Ciriolo, S. Di Guida, M. E. Dinardo, S. Fiorendi, S. Gennai, A. Ghezzi, P. Govoni, M. Malberti, S. Malvezzi, D. Menasce, F. Monti, L. Moroni, M. Paganoni, D. Pedrini, S. Ragazzi, T. Tabarelli de Fatis, D. Zuolo, S. Buontempo, N. Cavallo, A. De Iorio, A. Di Crescenzo, F. Fabozzi, F. Fienga, G. Galati, A. O. M. Iorio, L. Lista, S. Meola, P. Paolucci, C. Sciacca, E. Voevodina, P. Azzi, N. Bacchetta, D. Bisello, A. Boletti, A. Bragagnolo, R. Carlin, P. Checchia, M. Dall’Osso, P. De Castro Manzano, T. Dorigo, U. Dosselli, F. Gasparini, U. Gasparini, A. Gozzelino, S. Y. Hoh, S. Lacaprara, P. Lujan, M. Margoni, A. T. Meneguzzo, J. Pazzini, M. Presilla, P. Ronchese, R. Rossin, F. Simonetto, A. Tiko, E. Torassa, M. Tosi, M. Zanetti, P. Zotto, G. Zumerle, A. Braghieri, A. Magnani, P. Montagna, S. P. Ratti, V. Re, M. Ressegotti, C. Riccardi, P. Salvini, I. Vai, P. Vitulo, M. Biasini, G. M. Bilei, C. Cecchi, D. Ciangottini, L. Fanò, P. Lariccia, R. Leonardi, E. Manoni, G. Mantovani, V. Mariani, M. Menichelli, A. Rossi, A. Santocchia, D. Spiga, K. Androsov, P. Azzurri, G. Bagliesi, L. Bianchini, T. Boccali, L. Borrello, R. Castaldi, M. A. Ciocci, R. Dell’Orso, G. Fedi, F. Fiori, L. Giannini, A. Giassi, M. T. Grippo, F. Ligabue, E. Manca, G. Mandorli, A. Messineo, F. Palla, A. Rizzi, G. Rolandi, P. Spagnolo, R. Tenchini, G. Tonelli, A. Venturi, P. G. Verdini, L. Barone, F. Cavallari, M. Cipriani, D. Del Re, E. Di Marco, M. Diemoz, S. Gelli, E. Longo, B. Marzocchi, P. Meridiani, G. Organtini, F. Pandolfi, R. Paramatti, F. Preiato, S. Rahatlou, C. Rovelli, F. Santanastasio, N. Amapane, R. Arcidiacono, S. Argiro, M. Arneodo, N. Bartosik, R. Bellan, C. Biino, A. Cappati, N. Cartiglia, F. Cenna, S. Cometti, M. Costa, R. Covarelli, N. Demaria, B. Kiani, C. Mariotti, S. Maselli, E. Migliore, V. Monaco, E. Monteil, M. Monteno, M. M. Obertino, L. Pacher, N. Pastrone, M. Pelliccioni, G. L. Pinna Angioni, A. Romero, M. Ruspa, R. Sacchi, R. Salvatico, K. Shchelina, V. Sola, A. Solano, D. Soldi, A. Staiano, S. Belforte, V. Candelise, M. Casarsa, F. Cossutti, A. Da Rold, G. Della Ricca, F. Vazzoler, A. Zanetti, D. H. Kim, G. N. Kim, M. S. Kim, J. Lee, S. Lee, S. W. Lee, C. S. Moon, Y. D. Oh, S. I. Pak, S. Sekmen, D. C. Son, Y. C. Yang, H. Kim, D. H. Moon, G. Oh, B. Francois, J. Goh, T. J. Kim, S. Cho, S. Choi, Y. Go, D. Gyun, S. Ha, B. Hong, Y. Jo, K. Lee, K. S. Lee, S. Lee, J. Lim, S. K. Park, Y. Roh, H. S. Kim, J. Almond, J. Kim, J. S. Kim, H. Lee, K. Lee, K. Nam, S. B. Oh, B. C. Radburn-Smith, S. h. Seo, U. K. Yang, H. D. Yoo, G. B. Yu, D. Jeon, H. Kim, J. H. Kim, J. S. H. Lee, I. C. Park, Y. Choi, C. Hwang, J. Lee, I. Yu, V. Veckalns, V. Dudenas, A. Juodagalvis, J. Vaitkus, Z. A. Ibrahim, M. A. B. Md Ali, F. Mohamad Idris, W. A. T. Wan Abdullah, M. N. Yusli, Z. Zolkapli, J. F. Benitez, A. Castaneda Hernandez, J. A. Murillo Quijada, H. Castilla-Valdez, E. De La Cruz-Burelo, M. C. Duran-Osuna, I. Heredia-De La Cruz, R. Lopez-Fernandez, J. Mejia Guisao, R. I. Rabadan-Trejo, M. Ramirez-Garcia, G. Ramirez-Sanchez, R. Reyes-Almanza, A. Sanchez-Hernandez, S. Carrillo Moreno, C. Oropeza Barrera, F. Vazquez Valencia, J. Eysermans, I. Pedraza, H. A. Salazar Ibarguen, C. Uribe Estrada, A. Morelos Pineda, D. Krofcheck, S. Bheesette, P. H. Butler, A. Ahmad, M. Ahmad, M. I. Asghar, Q. Hassan, H. R. Hoorani, W. A. Khan, M. A. Shah, M. Shoaib, M. Waqas, H. Bialkowska, M. Bluj, B. Boimska, T. Frueboes, M. Górski, M. Kazana, M. Szleper, P. Traczyk, P. Zalewski, K. Bunkowski, A. Byszuk, K. Doroba, A. Kalinowski, M. Konecki, J. Krolikowski, M. Misiura, M. Olszewski, A. Pyskir, M. Walczak, M. Araujo, P. Bargassa, C. Beirão Da Cruz E Silva, A. Di Francesco, P. Faccioli, B. Galinhas, M. Gallinaro, J. Hollar, N. Leonardo, J. Seixas, G. Strong, O. Toldaiev, J. Varela, S. Afanasiev, P. Bunin, M. Gavrilenko, I. Golutvin, I. Gorbunov, A. Kamenev, V. Karjavine, A. Lanev, A. Malakhov, V. Matveev, P. Moisenz, V. Palichik, V. Perelygin, S. Shmatov, S. Shulha, N. Skatchkov, V. Smirnov, N. Voytishin, A. Zarubin, V. Golovtsov, Y. Ivanov, V. Kim, E. Kuznetsova, P. Levchenko, V. Murzin, V. Oreshkin, I. Smirnov, D. Sosnov, V. Sulimov, L. Uvarov, S. Vavilov, A. Vorobyev, Yu. Andreev, A. Dermenev, S. Gninenko, N. Golubev, A. Karneyeu, M. Kirsanov, N. Krasnikov, A. Pashenkov, A. Shabanov, D. Tlisov, A. Toropin, V. Epshteyn, V. Gavrilov, N. Lychkovskaya, V. Popov, I. Pozdnyakov, G. Safronov, A. Spiridonov, A. Stepennov, V. Stolin, M. Toms, E. Vlasov, A. Zhokin, T. Aushev, R. Chistov, M. Danilov, P. Parygin, E. Tarkovskii, V. Andreev, M. Azarkin, I. Dremin, M. Kirakosyan, A. Terkulov, A. Baskakov, A. Belyaev, E. Boos, V. Bunichev, M. Dubinin, L. Dudko, V. Klyukhin, N. Korneeva, I. Lokhtin, S. Obraztsov, M. Perfilov, V. Savrin, P. Volkov, A. Barnyakov, V. Blinov, T. Dimova, L. Kardapoltsev, Y. Skovpen, I. Azhgirey, I. Bayshev, S. Bitioukov, V. Kachanov, A. Kalinin, D. Konstantinov, P. Mandrik, V. Petrov, R. Ryutin, S. Slabospitskii, A. Sobol, S. Troshin, N. Tyurin, A. Uzunian, A. Volkov, A. Babaev, S. Baidali, V. Okhotnikov, P. Adzic, P. Cirkovic, D. Devetak, M. Dordevic, P. Milenovic, J. Milosevic, J. Alcaraz Maestre, A. Álvarez Fernández, I. Bachiller, M. Barrio Luna, J. A. Brochero Cifuentes, M. Cerrada, N. Colino, B. De La Cruz, A. Delgado Peris, C. Fernandez Bedoya, J. P. Fernández Ramos, J. Flix, M. C. Fouz, O. Gonzalez Lopez, S. Goy Lopez, J. M. Hernandez, M. I. Josa, D. Moran, A. Pérez-Calero Yzquierdo, J. Puerta Pelayo, I. Redondo, L. Romero, S. Sánchez Navas, M. S. Soares, A. Triossi, C. Albajar, J. F. de Trocóniz, J. Cuevas, C. Erice, J. Fernandez Menendez, S. Folgueras, I. Gonzalez Caballero, J. R. González Fernández, E. Palencia Cortezon, V. Rodríguez Bouza, S. Sanchez Cruz, J. M. Vizan Garcia, I. J. Cabrillo, A. Calderon, B. Chazin Quero, J. Duarte Campderros, M. Fernandez, P. J. Fernández Manteca, A. García Alonso, J. Garcia-Ferrero, G. Gomez, A. Lopez Virto, J. Marco, C. Martinez Rivero, P. Martinez Ruiz del Arbol, F. Matorras, J. Piedra Gomez, C. Prieels, T. Rodrigo, A. Ruiz-Jimeno, L. Scodellaro, N. Trevisani, I. Vila, R. Vilar Cortabitarte, N. Wickramage, D. Abbaneo, B. Akgun, E. Auffray, G. Auzinger, P. Baillon, A. H. Ball, D. Barney, J. Bendavid, M. Bianco, A. Bocci, C. Botta, E. Brondolin, T. Camporesi, M. Cepeda, G. Cerminara, E. Chapon, Y. Chen, G. Cucciati, D. d’Enterria, A. Dabrowski, N. Daci, V. Daponte, A. David, A. De Roeck, N. Deelen, M. Dobson, M. Dünser, N. Dupont, A. Elliott-Peisert, F. Fallavollita, D. Fasanella, G. Franzoni, J. Fulcher, W. Funk, D. Gigi, A. Gilbert, K. Gill, F. Glege, M. Gruchala, M. Guilbaud, D. Gulhan, J. Hegeman, C. Heidegger, V. Innocente, G. M. Innocenti, A. Jafari, P. Janot, O. Karacheban, J. Kieseler, A. Kornmayer, M. Krammer, C. Lange, P. Lecoq, C. Lourenço, L. Malgeri, M. Mannelli, A. Massironi, F. Meijers, J. A. Merlin, S. Mersi, E. Meschi, F. Moortgat, M. Mulders, J. Ngadiuba, S. Nourbakhsh, S. Orfanelli, L. Orsini, F. Pantaleo, L. Pape, E. Perez, M. Peruzzi, A. Petrilli, G. Petrucciani, A. Pfeiffer, M. Pierini, F. M. Pitters, D. Rabady, A. Racz, M. Rovere, H. Sakulin, C. Schäfer, C. Schwick, M. Selvaggi, A. Sharma, P. Silva, P. Sphicas, A. Stakia, J. Steggemann, D. Treille, A. Tsirou, A. Vartak, M. Verzetti, W. D. Zeuner, L. Caminada, K. Deiters, W. Erdmann, R. Horisberger, Q. Ingram, H. C. Kaestli, D. Kotlinski, U. Langenegger, T. Rohe, S. A. Wiederkehr, M. Backhaus, L. Bäni, P. Berger, N. Chernyavskaya, G. Dissertori, M. Dittmar, M. Donegà, C. Dorfer, T. A. Gómez Espinosa, C. Grab, D. Hits, T. Klijnsma, W. Lustermann, R. A. Manzoni, M. Marionneau, M. T. Meinhard, F. Micheli, P. Musella, F. Nessi-Tedaldi, F. Pauss, G. Perrin, L. Perrozzi, S. Pigazzini, M. Reichmann, C. Reissel, D. Ruini, D. A. Sanz Becerra, M. Schönenberger, L. Shchutska, V. R. Tavolaro, K. Theofilatos, M. L. Vesterbacka Olsson, R. Wallny, D. H. Zhu, T. K. Aarrestad, C. Amsler, D. Brzhechko, M. F. Canelli, A. De Cosa, R. Del Burgo, S. Donato, C. Galloni, T. Hreus, B. Kilminster, S. Leontsinis, I. Neutelings, G. Rauco, P. Robmann, D. Salerno, K. Schweiger, C. Seitz, Y. Takahashi, A. Zucchetta, T. H. Doan, R. Khurana, C. M. Kuo, W. Lin, S. S. Yu, P. Chang, Y. Chao, K. F. Chen, P. H. Chen, W.-S. Hou, Y. F. Liu, R.-S. Lu, E. Paganis, A. Psallidas, A. Steen, B. Asavapibhop, N. Srimanobhas, N. Suwonjandee, A. Bat, F. Boran, S. Cerci, S. Damarseckin, Z. S. Demiroglu, F. Dolek, C. Dozen, I. Dumanoglu, G. Gokbulut, Y. Guler, E. Gurpinar, I. Hos, C. Isik, E. E. Kangal, O. Kara, A. Kayis Topaksu, U. Kiminsu, M. Oglakci, G. Onengut, K. Ozdemir, S. Ozturk, D. Sunar Cerci, B. Tali, U. G. Tok, S. Turkcapar, I. S. Zorbakir, C. Zorbilmez, B. Isildak, G. Karapinar, M. Yalvac, M. Zeyrek, I. O. Atakisi, E. Gülmez, M. Kaya, O. Kaya, S. Ozkorucuklu, S. Tekten, E. A. Yetkin, M. N. Agaras, A. Cakir, K. Cankocak, Y. Komurcu, S. Sen, B. Grynyov, L. Levchuk, F. Ball, J. J. Brooke, D. Burns, E. Clement, D. Cussans, O. Davignon, H. Flacher, J. Goldstein, G. P. Heath, H. F. Heath, L. Kreczko, D. M. Newbold, S. Paramesvaran, B. Penning, T. Sakuma, D. Smith, V. J. Smith, J. Taylor, A. Titterton, K. W. Bell, A. Belyaev, C. Brew, R. M. Brown, D. Cieri, D. J. A. Cockerill, J. A. Coughlan, K. Harder, S. Harper, J. Linacre, K. Manolopoulos, E. Olaiya, D. Petyt, T. Reis, T. Schuh, C. H. Shepherd-Themistocleous, A. Thea, I. R. Tomalin, T. Williams, W. J. Womersley, R. Bainbridge, P. Bloch, J. Borg, S. Breeze, O. Buchmuller, A. Bundock, D. Colling, P. Dauncey, G. Davies, M. Della Negra, R. Di Maria, P. Everaerts, G. Hall, G. Iles, T. James, M. Komm, C. Laner, L. Lyons, A.-M. Magnan, S. Malik, A. Martelli, J. Nash, A. Nikitenko, V. Palladino, M. Pesaresi, D. M. Raymond, A. Richards, A. Rose, E. Scott, C. Seez, A. Shtipliyski, G. Singh, M. Stoye, T. Strebler, S. Summers, A. Tapper, K. Uchida, T. Virdee, N. Wardle, D. Winterbottom, J. Wright, S. C. Zenz, J. E. Cole, P. R. Hobson, A. Khan, P. Kyberd, C. K. Mackay, A. Morton, I. D. Reid, L. Teodorescu, S. Zahid, K. Call, J. Dittmann, K. Hatakeyama, H. Liu, C. Madrid, B. McMaster, N. Pastika, C. Smith, R. Bartek, A. Dominguez, A. Buccilli, S. I. Cooper, C. Henderson, P. Rumerio, C. West, D. Arcaro, T. Bose, Z. Demiragli, D. Gastler, S. Girgis, D. Pinna, C. Richardson, J. Rohlf, D. Sperka, I. Suarez, L. Sulak, D. Zou, G. Benelli, B. Burkle, X. Coubez, D. Cutts, M. Hadley, J. Hakala, U. Heintz, J. M. Hogan, K. H. M. Kwok, E. Laird, G. Landsberg, J. Lee, Z. Mao, M. Narain, S. Sagir, R. Syarif, E. Usai, D. Yu, R. Band, C. Brainerd, R. Breedon, D. Burns, M. Calderon De La Barca Sanchez, M. Chertok, J. Conway, R. Conway, P. T. Cox, R. Erbacher, C. Flores, G. Funk, W. Ko, O. Kukral, R. Lander, M. Mulhearn, D. Pellett, J. Pilot, S. Shalhout, M. Shi, D. Stolp, D. Taylor, K. Tos, M. Tripathi, Z. Wang, F. Zhang, M. Bachtis, C. Bravo, R. Cousins, A. Dasgupta, S Erhan, A. Florent, J. Hauser, M. Ignatenko, N. Mccoll, S. Regnard, D. Saltzberg, C. Schnaible, V. Valuev, E. Bouvier, K. Burt, R. Clare, J. W. Gary, S. M. A. Ghiasi Shirazi, G. Hanson, G. Karapostoli, E. Kennedy, F. Lacroix, O. R. Long, M. Olmedo Negrete, M. I. Paneva, W. Si, L. Wang, H. Wei, S. Wimpenny, B. R. Yates, J. G. Branson, P. Chang, S. Cittolin, M. Derdzinski, R. Gerosa, D. Gilbert, B. Hashemi, A. Holzner, D. Klein, G. Kole, V. Krutelyov, J. Letts, M. Masciovecchio, S. May, D. Olivito, S. Padhi, M. Pieri, V. Sharma, M. Tadel, J. Wood, F. Würthwein, A. Yagil, G. Zevi Della Porta, N. Amin, R. Bhandari, C. Campagnari, M. Citron, V. Dutta, M. Franco Sevilla, L. Gouskos, R. Heller, J. Incandela, H. Mei, A. Ovcharova, H. Qu, J. Richman, D. Stuart, S. Wang, J. Yoo, D. Anderson, A. Bornheim, J. M. Lawhorn, N. Lu, H. B. Newman, T. Q. Nguyen, J. Pata, M. Spiropulu, J. R. Vlimant, R. Wilkinson, S. Xie, Z. Zhang, R. Y. Zhu, M. B. Andrews, T. Ferguson, T. Mudholkar, M. Paulini, M. Sun, I. Vorobiev, M. Weinberg, J. P. Cumalat, W. T. Ford, F. Jensen, A. Johnson, E. MacDonald, T. Mulholland, R. Patel, A. Perloff, K. Stenson, K. A. Ulmer, S. R. Wagner, J. Alexander, J. Chaves, Y. Cheng, J. Chu, A. Datta, K. Mcdermott, N. Mirman, J. R. Patterson, D. Quach, A. Rinkevicius, A. Ryd, L. Skinnari, L. Soffi, S. M. Tan, Z. Tao, J. Thom, J. Tucker, P. Wittich, M. Zientek, S. Abdullin, M. Albrow, M. Alyari, G. Apollinari, A. Apresyan, A. Apyan, S. Banerjee, L. A. T. Bauerdick, A. Beretvas, J. Berryhill, P. C. Bhat, K. Burkett, J. N. Butler, A. Canepa, G. B. Cerati, H. W. K. Cheung, F. Chlebana, M. Cremonesi, J. Duarte, V. D. Elvira, J. Freeman, Z. Gecse, E. Gottschalk, L. Gray, D. Green, S. Grünendahl, O. Gutsche, J. Hanlon, R. M. Harris, S. Hasegawa, J. Hirschauer, Z. Hu, B. Jayatilaka, S. Jindariani, M. Johnson, U. Joshi, B. Klima, M. J. Kortelainen, B. Kreis, S. Lammel, D. Lincoln, R. Lipton, M. Liu, T. Liu, J. Lykken, K. Maeshima, J. M. Marraffino, D. Mason, P. McBride, P. Merkel, S. Mrenna, S. Nahn, V. O’Dell, K. Pedro, C. Pena, O. Prokofyev, G. Rakness, F. Ravera, A. Reinsvold, L. Ristori, A. Savoy-Navarro, B. Schneider, E. Sexton-Kennedy, A. Soha, W. J. Spalding, L. Spiegel, S. Stoynev, J. Strait, N. Strobbe, L. Taylor, S. Tkaczyk, N. V. Tran, L. Uplegger, E. W. Vaandering, C. Vernieri, M. Verzocchi, R. Vidal, M. Wang, H. A. Weber, D. Acosta, P. Avery, P. Bortignon, D. Bourilkov, A. Brinkerhoff, L. Cadamuro, A. Carnes, D. Curry, R. D. Field, S. V. Gleyzer, B. M. Joshi, J. Konigsberg, A. Korytov, K. H. Lo, P. Ma, K. Matchev, N. Menendez, G. Mitselmakher, D. Rosenzweig, K. Shi, J. Wang, S. Wang, X. Zuo, Y. R. Joshi, S. Linn, A. Ackert, T. Adams, A. Askew, S. Hagopian, V. Hagopian, K. F. Johnson, T. Kolberg, G. Martinez, T. Perry, H. Prosper, A. Saha, C. Schiber, R. Yohay, M. M. Baarmand, V. Bhopatkar, S. Colafranceschi, M. Hohlmann, D. Noonan, M. Rahmani, T. Roy, M. Saunders, F. Yumiceva, M. R. Adams, L. Apanasevich, D. Berry, R. R. Betts, R. Cavanaugh, X. Chen, S. Dittmer, O. Evdokimov, C. E. Gerber, D. A. Hangal, D. J. Hofman, K. Jung, J. Kamin, C. Mills, M. B. Tonjes, N. Varelas, H. Wang, X. Wang, Z. Wu, J. Zhang, M. Alhusseini, B. Bilki, W. Clarida, K. Dilsiz, S. Durgut, R. P. Gandrajula, M. Haytmyradov, V. Khristenko, J.-P. Merlo, A. Mestvirishvili, A. Moeller, J. Nachtman, H. Ogul, Y. Onel, F. Ozok, A. Penzo, C. Snyder, E. Tiras, J. Wetzel, B. Blumenfeld, A. Cocoros, N. Eminizer, D. Fehling, L. Feng, A. V. Gritsan, W. T. Hung, P. Maksimovic, J. Roskes, U. Sarica, M. Swartz, M. Xiao, A. Al-bataineh, P. Baringer, A. Bean, S. Boren, J. Bowen, A. Bylinkin, J. Castle, S. Khalil, A. Kropivnitskaya, D. Majumder, W. Mcbrayer, M. Murray, C. Rogan, S. Sanders, E. Schmitz, J. D. Tapia Takaki, Q. Wang, S. Duric, A. Ivanov, K. Kaadze, D. Kim, Y. Maravin, D. R. Mendis, T. Mitchell, A. Modak, A. Mohammadi, F. Rebassoo, D. Wright, A. Baden, O. Baron, A. Belloni, S. C. Eno, Y. Feng, C. Ferraioli, N. J. Hadley, S. Jabeen, G. Y. Jeng, R. G. Kellogg, J. Kunkle, A. C. Mignerey, S. Nabili, F. Ricci-Tam, M. Seidel, Y. H. Shin, A. Skuja, S. C. Tonwar, K. Wong, D. Abercrombie, B. Allen, V. Azzolini, A. Baty, R. Bi, S. Brandt, W. Busza, I. A. Cali, M. D’Alfonso, G. Gomez Ceballos, M. Goncharov, P. Harris, D. Hsu, M. Hu, Y. Iiyama, M. Klute, D. Kovalskyi, Y.-J. Lee, P. D. Luckey, B. Maier, A. C. Marini, C. Mcginn, C. Mironov, S. Narayanan, X. Niu, C. Paus, D. Rankin, C. Roland, G. Roland, Z. Shi, G. S. F. Stephans, K. Sumorok, K. Tatar, D. Velicanu, J. Wang, T. W. Wang, B. Wyslouch, A. C. Benvenuti, R. M. Chatterjee, A. Evans, P. Hansen, J. Hiltbrand, Sh. Jain, S. Kalafut, M. Krohn, Y. Kubota, Z. Lesko, J. Mans, R. Rusack, M. A. Wadud, J. G. Acosta, S. Oliveros, E. Avdeeva, K. Bloom, D. R. Claes, C. Fangmeier, F. Golf, R. Gonzalez Suarez, R. Kamalieddin, I. Kravchenko, J. Monroy, J. E. Siado, G. R. Snow, B. Stieger, A. Godshalk, C. Harrington, I. Iashvili, A. Kharchilava, C. Mclean, D. Nguyen, A. Parker, S. Rappoccio, B. Roozbahani, G. Alverson, E. Barberis, C. Freer, Y. Haddad, A. Hortiangtham, G. Madigan, D. M. Morse, T. Orimoto, A. Tishelman-charny, T. Wamorkar, B. Wang, A. Wisecarver, D. Wood, S. Bhattacharya, J. Bueghly, O. Charaf, T. Gunter, K. A. Hahn, N. Odell, M. H. Schmitt, K. Sung, M. Trovato, M. Velasco, R. Bucci, N. Dev, R. Goldouzian, M. Hildreth, K. Hurtado Anampa, C. Jessop, D. J. Karmgard, K. Lannon, W. Li, N. Loukas, N. Marinelli, F. Meng, C. Mueller, Y. Musienko, M. Planer, R. Ruchti, P. Siddireddy, G. Smith, S. Taroni, M. Wayne, A. Wightman, M. Wolf, A. Woodard, J. Alimena, L. Antonelli, B. Bylsma, L. S. Durkin, S. Flowers, B. Francis, C. Hill, W. Ji, T. Y. Ling, W. Luo, B. L. Winer, S. Cooperstein, G. Dezoort, P. Elmer, J. Hardenbrook, N. Haubrich, S. Higginbotham, A. Kalogeropoulos, S. Kwan, D. Lange, M. T. Lucchini, J. Luo, D. Marlow, K. Mei, I. Ojalvo, J. Olsen, C. Palmer, P. Piroué, J. Salfeld-Nebgen, D. Stickland, C. Tully, S. Malik, S. Norberg, A. Barker, V. E. Barnes, S. Das, L. Gutay, M. Jones, A. W. Jung, A. Khatiwada, B. Mahakud, D. H. Miller, N. Neumeister, C. C. Peng, S. Piperov, H. Qiu, J. F. Schulte, J. Sun, F. Wang, R. Xiao, W. Xie, T. Cheng, J. Dolen, N. Parashar, Z. Chen, K. M. Ecklund, S. Freed, F. J. M. Geurts, M. Kilpatrick, Arun Kumar, W. Li, B. P. Padley, R. Redjimi, J. Roberts, J. Rorie, W. Shi, Z. Tu, A. Zhang, A. Bodek, P. de Barbaro, R. Demina, Y. t. Duh, J. L. Dulemba, C. Fallon, T. Ferbel, M. Galanti, A. Garcia-Bellido, J. Han, O. Hindrichs, A. Khukhunaishvili, E. Ranken, P. Tan, R. Taus, B. Chiarito, J. P. Chou, Y. Gershtein, E. Halkiadakis, A. Hart, M. Heindl, E. Hughes, S. Kaplan, R. Kunnawalkam Elayavalli, S. Kyriacou, I. Laflotte, A. Lath, R. Montalvo, K. Nash, M. Osherson, H. Saka, S. Salur, S. Schnetzer, D. Sheffield, S. Somalwar, R. Stone, S. Thomas, P. Thomassen, H. Acharya, A. G. Delannoy, J. Heideman, G. Riley, S. Spanier, O. Bouhali, A. Celik, M. Dalchenko, M. De Mattia, A. Delgado, S. Dildick, R. Eusebi, J. Gilmore, T. Huang, T. Kamon, S. Luo, D. Marley, R. Mueller, D. Overton, L. Perniè, D. Rathjens, A. Safonov, N. Akchurin, J. Damgov, F. De Guio, P. R. Dudero, S. Kunori, K. Lamichhane, S. W. Lee, T. Mengke, S. Muthumuni, T. Peltola, S. Undleeb, I. Volobouev, Z. Wang, A. Whitbeck, S. Greene, A. Gurrola, R. Janjam, W. Johns, C. Maguire, A. Melo, H. Ni, K. Padeken, F. Romeo, P. Sheldon, S. Tuo, J. Velkovska, M. Verweij, Q. Xu, M. W. Arenton, P. Barria, B. Cox, R. Hirosky, M. Joyce, A. Ledovskoy, H. Li, C. Neu, T. Sinthuprasith, Y. Wang, E. Wolfe, F. Xia, R. Harr, P. E. Karchin, N. Poudyal, J. Sturdy, P. Thapa, S. Zaleski, J. Buchanan, C. Caillol, D. Carlsmith, S. Dasu, I. De Bruyn, L. Dodd, B. Gomber, M. Grothe, M. Herndon, A. Hervé, U. Hussain, P. Klabbers, A. Lanaro, K. Long, R. Loveless, T. Ruggles, A. Savin, V. Sharma, N. Smith, W. H. Smith, N. Woods, CMS Collaboration

**Affiliations:** 1 Yerevan Physics Institute, Yerevan, Armenia; 2 Institut für Hochenergiephysik, Wien, Austria; 3 Institute for Nuclear Problems, Minsk, Belarus; 4 Universiteit Antwerpen, Antwerpen, Belgium; 5 Vrije Universiteit Brussel, Brussels, Belgium; 6 Université Libre de Bruxelles, Brussels, Belgium; 7 Ghent University, Ghent, Belgium; 8 Université Catholique de Louvain, Louvain-la-Neuve, Belgium; 9 Centro Brasileiro de Pesquisas Fisicas, Rio de Janeiro, Brazil; 10 Universidade do Estado do Rio de Janeiro, Rio de Janeiro, Brazil; 11 Universidade Estadual Paulista , Universidade Federal do ABC, São Paulo, Brazil; 12 Institute for Nuclear Research and Nuclear Energy, Bulgarian Academy of Sciences, Sofia, Bulgaria; 13 University of Sofia, Sofia, Bulgaria; 14 Beihang University, Beijing, China; 15 Institute of High Energy Physics, Beijing, China; 16 State Key Laboratory of Nuclear Physics and Technology, Peking University, Beijing, China; 17 Tsinghua University, Beijing, China; 18 Universidad de Los Andes, Bogotá, Colombia; 19 Faculty of Electrical Engineering, Mechanical Engineering and Naval Architecture, University of Split, Split, Croatia; 20 Faculty of Science, University of Split, Split, Croatia; 21 Institute Rudjer Boskovic, Zagreb, Croatia; 22 University of Cyprus, Nicosia, Cyprus; 23 Charles University, Prague, Czech Republic; 24 Escuela Politecnica Nacional, Quito, Ecuador; 25 Universidad San Francisco de Quito, Quito, Ecuador; 26 Academy of Scientific Research and Technology of the Arab Republic of Egypt, Egyptian Network of High Energy Physics, Cairo, Egypt; 27 National Institute of Chemical Physics and Biophysics, Tallinn, Estonia; 28 Department of Physics, University of Helsinki, Helsinki, Finland; 29 Helsinki Institute of Physics, Helsinki, Finland; 30 Lappeenranta University of Technology, Lappeenranta, Finland; 31 IRFU, CEA, Université Paris-Saclay, Gif-sur-Yvette, France; 32 Laboratoire Leprince-Ringuet, Ecole polytechnique, CNRS/IN2P3, Université Paris-Saclay, Palaiseau, France; 33 Université de Strasbourg, CNRS, IPHC UMR 7178, Strasbourg, France; 34 Centre de Calcul de l’Institut National de Physique Nucleaire et de Physique des Particules, CNRS/IN2P3, Villeurbanne, France; 35 Université de Lyon, Université Claude Bernard Lyon 1, CNRS-IN2P3, Institut de Physique Nucléaire de Lyon, Villeurbanne, France; 36 Georgian Technical University, Tbilisi, Georgia; 37 Tbilisi State University, Tbilisi, Georgia; 38 RWTH Aachen University, I. Physikalisches Institut, Aachen, Germany; 39 RWTH Aachen University, III. Physikalisches Institut A, Aachen, Germany; 40 RWTH Aachen University, III. Physikalisches Institut B, Aachen, Germany; 41 Deutsches Elektronen-Synchrotron, Hamburg, Germany; 42 University of Hamburg, Hamburg, Germany; 43 Karlsruher Institut fuer Technologie, Karlsruhe, Germany; 44 Institute of Nuclear and Particle Physics (INPP), NCSR Demokritos, Aghia Paraskevi, Greece; 45 National and Kapodistrian University of Athens, Athens, Greece; 46 National Technical University of Athens, Athens, Greece; 47 University of Ioánnina, Ioannina, Greece; 48 MTA-ELTE Lendület CMS Particle and Nuclear Physics Group, Eötvös Loránd University, Budapest, Hungary; 49 Wigner Research Centre for Physics, Budapest, Hungary; 50 Institute of Nuclear Research ATOMKI, Debrecen, Hungary; 51 Institute of Physics, University of Debrecen, Debrecen, Hungary; 52 Indian Institute of Science (IISc), Bangalore, India; 53 National Institute of Science Education and Research, HBNI, Bhubaneswar, India; 54 Panjab University, Chandigarh, India; 55 University of Delhi, Delhi, India; 56 Saha Institute of Nuclear Physics, HBNI, Kolkata, India; 57 Indian Institute of Technology Madras, Madras, India; 58 Bhabha Atomic Research Centre, Mumbai, India; 59 Tata Institute of Fundamental Research-A, Mumbai, India; 60 Tata Institute of Fundamental Research-B, Mumbai, India; 61 Indian Institute of Science Education and Research (IISER), Pune, India; 62 Institute for Research in Fundamental Sciences (IPM), Tehran, Iran; 63 University College Dublin, Dublin, Ireland; 64 INFN Sezione di Bari, Università di Bari, Politecnico di Bari, Bari, Italy; 65 INFN Sezione di Bologna, Università di Bologna, Bologna, Italy; 66 INFN Sezione di Catania, Università di Catania, Catania, Italy; 67 INFN Sezione di Firenze, Università di Firenze, Firenze, Italy; 68 INFN Laboratori Nazionali di Frascati, Frascati, Italy; 69 INFN Sezione di Genova, Università di Genova, Genoa, Italy; 70 INFN Sezione di Milano-Bicocca, Università di Milano-Bicocca, Milan, Italy; 71 INFN Sezione di Napoli, Università di Napoli ’Federico II’ , Napoli, Italy, Università della Basilicata, Potenza, Italy, Università G. Marconi, Rome, Italy; 72 INFN Sezione di Padova, Università di Padova, Padova, Italy, Università di Trento, Trento, Italy; 73 INFN Sezione di Pavia, Università di Pavia, Pavia, Italy; 74 INFN Sezione di Perugia, Università di Perugia, Perugia, Italy; 75 INFN Sezione di Pisa, Università di Pisa, Scuola Normale Superiore di Pisa, Pisa, Italy; 76 INFN Sezione di Roma, Sapienza Università di Roma, Rome, Italy; 77 INFN Sezione di Torino, Università di Torino, Torino, Italy, Università del Piemonte Orientale, Novara, Italy; 78 INFN Sezione di Trieste, Università di Trieste, Trieste, Italy; 79 Kyungpook National University, Daegu, Korea; 80 Chonnam National University, Institute for Universe and Elementary Particles, Kwangju, Korea; 81 Hanyang University, Seoul, Korea; 82 Korea University, Seoul, Korea; 83 Sejong University, Seoul, Korea; 84 Seoul National University, Seoul, Korea; 85 University of Seoul, Seoul, Korea; 86 Sungkyunkwan University, Suwon, Korea; 87 Riga Technical University, Riga, Latvia; 88 Vilnius University, Vilnius, Lithuania; 89 National Centre for Particle Physics, Universiti Malaya, Kuala Lumpur, Malaysia; 90 Universidad de Sonora (UNISON), Hermosillo, Mexico; 91 Centro de Investigacion y de Estudios Avanzados del IPN, Mexico City, Mexico; 92 Universidad Iberoamericana, Mexico City, Mexico; 93 Benemerita Universidad Autonoma de Puebla, Puebla, Mexico; 94 Universidad Autónoma de San Luis Potosí, San Luis Potosí, Mexico; 95 University of Auckland, Auckland, New Zealand; 96 University of Canterbury, Christchurch, New Zealand; 97 National Centre for Physics, Quaid-I-Azam University, Islamabad, Pakistan; 98 National Centre for Nuclear Research, Swierk, Poland; 99 Institute of Experimental Physics, Faculty of Physics, University of Warsaw, Warsaw, Poland; 100 Laboratório de Instrumentação e Física Experimental de Partículas, Lisbon, Portugal; 101 Joint Institute for Nuclear Research, Dubna, Russia; 102 Petersburg Nuclear Physics Institute, Gatchina, St. Petersburg, Russia; 103 Institute for Nuclear Research, Moscow, Russia; 104 Institute for Theoretical and Experimental Physics, Moscow, Russia; 105 Moscow Institute of Physics and Technology, Moscow, Russia; 106 National Research Nuclear University ‘Moscow Engineering Physics Institute’ (MEPhI), Moscow, Russia; 107 P.N. Lebedev Physical Institute, Moscow, Russia; 108 Skobeltsyn Institute of Nuclear Physics, Lomonosov Moscow State University, Moscow, Russia; 109 Novosibirsk State University (NSU), Novosibirsk, Russia; 110 Institute for High Energy Physics of National Research Centre ‘Kurchatov Institute’, Protvino, Russia; 111 National Research Tomsk Polytechnic University, Tomsk, Russia; 112 Faculty of Physics and Vinca Institute of Nuclear Sciences, University of Belgrade, Belgrade, Serbia; 113 Centro de Investigaciones Energéticas Medioambientales y Tecnológicas (CIEMAT), Madrid, Spain; 114 Universidad Autónoma de Madrid, Madrid, Spain; 115 Universidad de Oviedo, Oviedo, Spain; 116 Instituto de Física de Cantabria (IFCA), CSIC-Universidad de Cantabria, Santander, Spain; 117 Department of Physics, University of Ruhuna, Matara, Sri Lanka; 118 CERN, European Organization for Nuclear Research, Geneva, Switzerland; 119 Paul Scherrer Institut, Villigen, Switzerland; 120 ETH Zurich-Institute for Particle Physics and Astrophysics (IPA), Zurich, Switzerland; 121 Universität Zürich, Zurich, Switzerland; 122 National Central University, Chung-Li, Taiwan; 123 National Taiwan University (NTU), Taipei, Taiwan; 124 Department of Physics, Faculty of Science, Chulalongkorn University, Bangkok, Thailand; 125 Physics Department, Science and Art Faculty, Çukurova University, Adana, Turkey; 126 Physics Department, Middle East Technical University, Ankara, Turkey; 127 Bogazici University, Istanbul, Turkey; 128 Istanbul Technical University, Istanbul, Turkey; 129 Institute for Scintillation Materials of National Academy of Science of Ukraine, Kharkov, Ukraine; 130 National Scientific Center, Kharkov Institute of Physics and Technology, Kharkov, Ukraine; 131 University of Bristol, Bristol, UK; 132 Rutherford Appleton Laboratory, Didcot, UK; 133 Imperial College, London, UK; 134 Brunel University, Uxbridge, UK; 135 Baylor University, Waco, USA; 136 Catholic University of America, Washington, DC USA; 137 The University of Alabama, Tuscaloosa, USA; 138 Boston University, Boston, USA; 139 Brown University, Providence, USA; 140 University of California, Davis, Davis, USA; 141 University of California, Los Angeles, USA; 142 University of California, Riverside, Riverside, USA; 143 University of California, San Diego, La Jolla, USA; 144 Department of Physics, University of California, Santa Barbara, Santa Barbara, USA; 145 California Institute of Technology, Pasadena, USA; 146 Carnegie Mellon University, Pittsburgh, USA; 147 University of Colorado Boulder, Boulder, USA; 148 Cornell University, Ithaca, USA; 149 Fermi National Accelerator Laboratory, Batavia, USA; 150 University of Florida, Gainesville, USA; 151 Florida International University, Miami, USA; 152 Florida State University, Tallahassee, USA; 153 Florida Institute of Technology, Melbourne, USA; 154 University of Illinois at Chicago (UIC), Chicago, USA; 155 The University of Iowa, Iowa City, USA; 156 Johns Hopkins University, Baltimore, USA; 157 The University of Kansas, Lawrence, USA; 158 Kansas State University, Manhattan, USA; 159 Lawrence Livermore National Laboratory, Livermore, USA; 160 University of Maryland, College Park, USA; 161 Massachusetts Institute of Technology, Cambridge, USA; 162 University of Minnesota, Minneapolis, USA; 163 University of Mississippi, Oxford, USA; 164 University of Nebraska-Lincoln, Lincoln, USA; 165 State University of New York at Buffalo, Buffalo, USA; 166 Northeastern University, Boston, USA; 167 Northwestern University, Evanston, USA; 168 University of Notre Dame, Notre Dame, USA; 169 The Ohio State University, Columbus, USA; 170 Princeton University, Princeton, USA; 171 University of Puerto Rico, Mayaguez, USA; 172 Purdue University, West Lafayette, USA; 173 Purdue University Northwest, Hammond, USA; 174 Rice University, Houston, USA; 175 University of Rochester, Rochester, USA; 176 Rutgers, The State University of New Jersey, Piscataway, USA; 177 University of Tennessee, Knoxville, USA; 178 Texas A&M University, College Station, USA; 179 Texas Tech University, Lubbock, USA; 180 Vanderbilt University, Nashville, USA; 181 University of Virginia, Charlottesville, USA; 182 Wayne State University, Detroit, USA; 183 University of Wisconsin-Madison, Madison, WI USA; 184 CERN, 1211 Geneva 23, Switzerland

## Abstract

A measurement of the top quark–antiquark pair production cross section $\sigma _{\mathrm {t}\overline{\mathrm {t}}}$ in proton–proton collisions at a centre-of-mass energy of 13$\,\text {Te}\text {V}$ is presented. The data correspond to an integrated luminosity of $35.9{\,\text {fb}^{-1}}$, recorded by the CMS experiment at the CERN LHC in 2016. Dilepton events ($\mathrm {e}$ $^{\pm }$ $\mathrm {\mu }$ $^{{\mp }}$, $\mathrm {\mu ^+}\mathrm {\mu ^-}$, $\mathrm {e}^+\mathrm {e}^-$) are selected and the cross section is measured from a likelihood fit. For a top quark mass parameter in the simulation of $m_\mathrm {\mathrm {t}} ^{\mathrm {MC}} = 172.5 \,\text {Ge}\text {V}$ the fit yields a measured cross section $\sigma _{\mathrm {t}\overline{\mathrm {t}}} = 803 \pm 2 \,\text {(stat)} \pm 25 \,\text {(syst)} \pm 20 \,\text {(lumi)} \,\text {pb}$, in agreement with the expectation from the standard model calculation at next-to-next-to-leading order. A simultaneous fit of the cross section and the top quark mass parameter in the powheg simulation is performed. The measured value of $m_\mathrm {\mathrm {t}} ^{\mathrm {MC}} = 172.33 \pm 0.14 \,\text {(stat)} \,^{+0.66}_{-0.72} \,\text {(syst)} \,\text {Ge}\text {V}$ is in good agreement with previous measurements. The resulting cross section is used, together with the theoretical prediction, to determine the top quark mass and to extract a value of the strong coupling constant with different sets of parton distribution functions.

## Introduction

Measurements of the top quark–antiquark pair cross section $\sigma _{\mathrm {t}\overline{\mathrm {t}}}$ in proton–proton (pp) collisions provide important tests of the standard model (SM). At the CERN LHC, measurements with increasing precision have been performed by the ATLAS and CMS Collaborations in several different decay channels and at four pp collision energies [1–5]. Precise theoretical predictions of $\sigma _{\mathrm {t}\overline{\mathrm {t}}}$ have been performed in perturbative quantum chromodynamics (QCD) at next-to-next-to-leading order (NNLO) [6–9]. The calculations depend on several fundamental parameters: the top quark mass $m_\mathrm {\mathrm {t}}$, the strong coupling constant $\alpha _S$, and the parton distribution functions (PDFs) of the proton. The measurements of $\sigma _{\mathrm {t}\overline{\mathrm {t}}}$ have been used to determine the top quark pole mass [1, 4, 10–12], $\alpha _S$  [4, 13], and the PDFs [14–17].

The value of $m_\mathrm {\mathrm {t}}$ significantly affects the prediction for many observables, either directly or via radiative corrections. It is a key input to electroweak precision fits [18] and, together with the value of the Higgs boson mass and $\alpha _S$, it has direct implications on the SM predictions for the stability of the electroweak vacuum [19]. In QCD calculations beyond leading order, $m_\mathrm {\mathrm {t}}$ depends on the renormalization scheme. In the context of the $\sigma _{\mathrm {t}\overline{\mathrm {t}}}$ predictions, the pole (on-shell) definition for the top quark mass $m_\mathrm {\mathrm {t}} ^{\text {pole}}$ has wide applications; however, it suffers from the renormalon problem that introduces a theoretical ambiguity in its definition. The minimal subtraction ($\mathrm {\overline{MS}}$) renormalization scheme has been shown to have a faster convergence than other schemes [20]. The relation between the pole and $\mathrm {\overline{MS}}$ masses is known to the four-loop level in QCD [21]. Experimentally, the most precise measurements of the top quark mass are obtained in so-called direct measurements performed at the Tevatron and LHC [22–25]. Except for a few cases such as Ref. [26], the measurements rely on Monte Carlo (MC) generators to provide the relation between the top quark mass and an experimental observable. Current MC generators implement matrix elements at leading or next-to-leading order (NLO), while higher orders are simulated through parton showering. Studies suggest that the top quark mass parameter $m_\mathrm {\mathrm {t}} ^{\mathrm {MC}}$, as implemented in current MC generators, corresponds to $m_\mathrm {\mathrm {t}} ^{\text {pole}}$ to an uncertainty on the order of 1$\,\text {Ge}\text {V}$  [27, 28]. A theoretically well-defined mass can be determined by comparing the measured ${\mathrm {t}\overline{\mathrm {t}}}$ cross section to the fixed-order theoretical predictions [1, 4, 10–12].

With the exception of the quark masses, $\alpha _S$ is the only free parameter in the QCD Lagrangian. While the renormalization group equation predicts the energy dependence of $\alpha _S$, i.e. it gives a functional form for $\alpha _S (Q)$, where *Q* is the energy scale of the process, actual values of $\alpha _S$ can only be obtained from experimental data. By convention and to facilitate comparisons, $\alpha _S$ values measured at different energy scales are typically evolved to $Q = m_\mathrm {\mathrm {Z}}$, the mass of the $\mathrm {Z}$ boson. The current world-average value for $\alpha _S (m_\mathrm {\mathrm {Z}})$ is $0.1181 \pm 0.0011$ [29]. In spite of this relatively precise result, the uncertainty in $\alpha _S$ still contributes significantly to many QCD predictions, including cross sections for top quark or Higgs boson production. Very few measurements allow $\alpha _S$ to be tested at high *Q*, and the precision on the world-average value for $\alpha _S (Q)$ is driven by low-*Q* measurements. A determination of $\sigma _{\mathrm {t}\overline{\mathrm {t}}}$ was used by the CMS Collaboration to extract the value of $\alpha _S (m_\mathrm {\mathrm {Z}})$ at NNLO for the first time [11]. In the prediction for $\sigma _{\mathrm {t}\overline{\mathrm {t}}}$, $\alpha _S$ appears not only in the expression for the parton-parton interaction but also in the QCD evolution of the PDFs. Varying the value of $\alpha _S (m_\mathrm {\mathrm {Z}})$ in the $\sigma _{\mathrm {t}\overline{\mathrm {t}}}$ calculation therefore requires a consistent modification of the PDFs. The full correlation between the gluon PDF, $\alpha _S$, and $m_\mathrm {\mathrm {t}}$ in the prediction for $\sigma _{\mathrm {t}\overline{\mathrm {t}}}$ has to be accounted for.

The analysis uses events in the dileptonic decay channels in which the two $\mathrm {W}$ bosons from the electroweak decays of the two top quarks each produce an electron or a muon, leading to three event categories: $\mathrm {e}$ $^{\pm }$ $\mathrm {\mu }$ $^{{\mp }}$, $\mathrm {\mu ^+}\mathrm {\mu ^-}$, and $\mathrm {e}^+\mathrm {e}^-$. The data set was recorded by CMS in 2016 at a centre-of-mass energy of 13$\,\text {Te}\text {V}$, corresponding to an integrated luminosity of $35.9{\,\text {fb}^{-1}}$. The measurement is performed using a maximum-likelihood fit in which the sources of systematic uncertainty are treated as nuisance parameters. Distributions of observables are chosen as input to the fit so as to further constrain the uncertainties. The fitting procedure largely follows the approach of Ref. [4]. In this analysis, the number of events is significantly larger than in previous data sets, thus providing tighter constraints. The dominant uncertainties come from the integrated luminosity and the efficiency to identify the two leptons. The correlation between the three decay channels is used to constrain the overall lepton identification uncertainty to that of the better-constrained lepton, which is the muon.

Experimentally, the measured value of $\sigma _{\mathrm {t}\overline{\mathrm {t}}}$ has a residual dependence on the value of $m_\mathrm {\mathrm {t}} ^{\mathrm {MC}}$ used in the simulation to estimate the detector efficiency and acceptance. In contrast, the experimental dependence of $\sigma _{\mathrm {t}\overline{\mathrm {t}}}$ on the value of $\alpha _S (m_\mathrm {\mathrm {Z}})$ used in the simulation is negligible [11]. For the extraction of a theoretically well-defined $m_\mathrm {\mathrm {t}}$, the dependence of the cross section on the assumption of a $m_\mathrm {\mathrm {t}} ^{\mathrm {MC}}$ value can be reduced by including $m_\mathrm {\mathrm {t}} ^{\mathrm {MC}}$ as an additional free parameter in the fit [30]. In this paper, the cross section $\sigma _{\mathrm {t}\overline{\mathrm {t}}}$ is first measured for a fixed value of $m_\mathrm {\mathrm {t}} ^{\mathrm {MC}} = 172.5 \,\text {Ge}\text {V}$, and then determined simultaneously with $m_\mathrm {\mathrm {t}} ^{\mathrm {MC}}$. In the simultaneous fit, input distributions sensitive to the top quark mass are introduced in order to constrain $m_\mathrm {\mathrm {t}} ^{\mathrm {MC}}$. For the measured parameter $m_\mathrm {\mathrm {t}} ^{\mathrm {MC}}$, the same systematic uncertainties are taken into account as in Ref. [31]. Finally, the measured value of $\sigma _{\mathrm {t}\overline{\mathrm {t}}}$ at the experimentally constrained value of $m_\mathrm {\mathrm {t}} ^{\mathrm {MC}}$ is used to extract $\alpha _S (m_\mathrm {\mathrm {Z}})$ and $m_\mathrm {\mathrm {t}}$ in the $\mathrm {\overline{MS}}$ scheme, using different PDF sets. For $m_\mathrm {\mathrm {t}}$, the pole mass scheme is also considered.

The paper is structured as follows. After a brief description of the CMS experiment and the MC event generators in Sect. 2, the event selection is presented in Sect. 3. The event categories and the maximum-likelihood fit are explained in Sect. 4. The systematic uncertainties in the measurement are discussed in Sect. 5. The result of the cross section measurement at a fixed value of $m_\mathrm {\mathrm {t}} ^{\mathrm {MC}} = 172.5 \,\text {Ge}\text {V}$ is presented in Sect. 6, and the simultaneous measurement of $\sigma _{\mathrm {t}\overline{\mathrm {t}}}$ and $m_\mathrm {\mathrm {t}} ^{\mathrm {MC}}$ is presented in Sect. 7. The extraction of $m_\mathrm {\mathrm {t}}$ and $\alpha _S$ in the $\mathrm {\overline{MS}}$ scheme and the top quark pole mass are described in Sects. 8 and 9, respectively, and a summary is given in Sect. 10.

## The CMS detector and Monte Carlo simulation

The central feature of the CMS apparatus [32] is a superconducting solenoid of 6$\,\text {m}$ internal diameter, providing a magnetic field of 3.8$\,\text {T}$. Within the solenoid volume are a silicon pixel and strip tracker, a lead tungstate crystal electromagnetic calorimeter, and a brass and scintillator hadron calorimeter, each composed of a barrel and two endcap sections. These are used to identify electrons, photons, and jets. Forward calorimeters extend the pseudorapidity coverage provided by the barrel and endcap detectors. Muons are detected in gas-ionization chambers embedded in the steel flux-return yoke outside the solenoid. The detector is nearly hermetic, providing reliable measurement of the momentum imbalance in the plane transverse to the beams. A two-level trigger system selects interesting events for offline analysis [33]. A more detailed description of the CMS detector, together with a definition of the coordinate system used and the relevant kinematic variables, can be found in Ref. [32].

The powheg  v2 [34–36] NLO MC generator is used to simulate ${\mathrm {t}\overline{\mathrm {t}}}$ events [37] and its model dependencies on $m_\mathrm {\mathrm {t}} ^{\mathrm {MC}}$, the PDFs [37], and the renormalization and factorization scales, $\mu _\mathrm {r} =\mu _\mathrm {f} =m_{\mathrm {T}} =\sqrt{\smash [b]{m_\mathrm {\mathrm {t}} ^2+p_{\mathrm {T}} ^2}}$, where $m_\mathrm {\mathrm {t}}$ is the pole mass and $p_{\mathrm {T}}$ is the transverse momentum of the top quark. The PDF set NNPDF3.0 [38] is used to describe the proton structure. The parton showers are modelled using pythia  8.2 [39] with the CUETP8M2T4 underlying event (UE) tune [40, 41]. In this analysis, ${\mathrm {t}\overline{\mathrm {t}}}$ events are split into a signal and a background component. The signal consists of dilepton events and includes contributions from leptonically decaying $\mathrm {\tau }$ leptons. All other ${\mathrm {t}\overline{\mathrm {t}}}$ events are considered as background.

Contributions to the background include single top quark processes ($\mathrm {t}\mathrm {W}$), Drell–Yan (DY) events ($\mathrm {Z}/\gamma ^*$+jets), and $\mathrm {W}$+jets production, as well as diboson (VV) events (including $\mathrm {W}$ $\mathrm {W}$, $\mathrm {W}$ $\mathrm {Z}$, and $\mathrm {Z}$ $\mathrm {Z}$) with multiple jets, while the contribution from QCD multijet production is found to be negligible. The DY and $\mathrm {t}\mathrm {W}$ processes are simulated in powheg  v2 [42–44] with the NNPDF3.0 PDF and interfaced to pythia  8.202 with the UE tune CUETP8M2T4 [45] for hadronization and fragmentation. The $\mathrm {W}$+jets events are generated at NLO using MadGraph 5_amc@nlo  2.2.2 [46, 47] with the NNPDF3.0 PDF and pythia  8.2 with the UE tune CUETP8M1. Events with $\mathrm {W}$ $\mathrm {W}$, $\mathrm {W}$ $\mathrm {Z}$, and $\mathrm {Z}$ $\mathrm {Z}$ diboson processes are generated at leading order using pythia  8.2 with the NNPDF2.3 PDF and the CUETP8M1 tune.

To model the effect of additional pp interactions within the same or nearby bunch crossing (pileup), simulated minimum bias interactions are added to the simulated data. Events in the simulation are then weighted to reproduce the pileup distribution in the data, which is estimated from the measured bunch-to-bunch instantaneous luminosity, assuming a total inelastic pp cross section of 69.2$\,\text {mb}$  [48].

For comparison with the measured distributions, the event yields in the simulated samples are normalized to their cross section predictions. These are obtained from calculations at NNLO (for $\mathrm {W}$+jets and $\mathrm {Z}/\gamma ^*$+jets [49]), NLO plus next-to-next-to-leading logarithms (NNLL) (for $\mathrm {t}\mathrm {W}$ production [50]), and NLO (for diboson processes [51]). For the simulated ${\mathrm {t}\overline{\mathrm {t}}}$ sample, the full NNLO+NNLL calculation, performed with the Top++  2.0 program, is used [52]. The proton structure is described by the CT14nnlo [53] PDF set, where the PDF and $\alpha _S$ uncertainties are estimated using the prescription by the authors. These are added in quadrature to the uncertainties originating from the scale variation $m_\mathrm {\mathrm {t}}/2<\mu _\mathrm {r}, \mu _\mathrm {f} <2m_\mathrm {\mathrm {t}}$. The cross section prediction is $\sigma _{{\mathrm {t}\overline{\mathrm {t}}}}^{\text {theo}} = 832~^{+20}_{-29}\, (\text {scale}) \pm 35 ~(\text {PDF}+\alpha _S ) \,\text {pb}$, assuming a top quark pole mass of 172.5$\,\text {Ge}\text {V}$.

## Event selection

Events with at least two leptons (electron or muon) of opposite charge are selected. In events with more than two leptons, the two leptons of opposite charge with the highest $p_{\mathrm {T}}$ are used. An event sample of three mutually exclusive event categories $\mathrm {e}$ $^{\pm }$ $\mathrm {\mu }$ $^{{\mp }}$, $\mathrm {\mu ^+}\mathrm {\mu ^-}$, and $\mathrm {e}^+\mathrm {e}^-$ is obtained.

A combination of single and dilepton triggers is used to collect the events. Each event is required to pass at least one of the triggers described below. Events in the $\mathrm {e}$ $^{\pm }$ $\mathrm {\mu }$ $^{{\mp }}$ channel are required to contain either one electron with $p_{\mathrm {T}} > 12\,\text {Ge}\text {V}$ and one muon with $p_{\mathrm {T}} > 23\,\text {Ge}\text {V}$, or one electron with $p_{\mathrm {T}} > 23\,\text {Ge}\text {V}$ and one muon with $p_{\mathrm {T}} > 8\,\text {Ge}\text {V}$. Events in the same-flavour channels are required to have $p_{\mathrm {T}} > 23\,(17)\,\text {Ge}\text {V}$ for the electron (muon) with the higher $p_{\mathrm {T}}$, referred to in the following as the leading lepton, and $p_{\mathrm {T}} > 12\,(8) \,\text {Ge}\text {V}$ for the other electron (muon), referred to as the subleading lepton. For all channels, single-lepton triggers with one electron (muon) with $p_{\mathrm {T}} > 27\,(24)\,\text {Ge}\text {V}$  are also used.

The particle-flow (PF) algorithm aims to reconstruct and identify each individual particle in an event, and to form PF candidates by combining information from the various components of the CMS detector [54]. The reconstructed vertex with the largest value of summed physics-object $p_{\mathrm {T}} ^2$ is taken to be the primary pp interaction vertex.

Electron and muon candidates are identified through their specific signatures in the detector [55, 56]. Lepton candidates are required to have $p_{\mathrm {T}} > 25\,(20)\,\text {Ge}\text {V}$ for the leading (subleading) lepton, in the range $|\eta | < 2.4$. Electron candidates in the transition region between the barrel and endcap calorimeters, corresponding to $1.4442< |\eta |< 1.5660$, are rejected because the reconstruction of electrons in this region is not optimal.

Lepton isolation requirements are based on the ratio of the scalar sum of the $p_{\mathrm {T}}$ of neighbouring PF candidates to the $p_{\mathrm {T}}$ of the lepton candidate, which is referred to as the lepton isolation variable. These PF candidates are the ones falling within a cone of size $\varDelta R=0.3 \,(0.4)$ for electrons (muons), centred on the lepton direction, excluding the contribution from the lepton candidate itself. The cone size $\varDelta R$ is defined as the square root of the quadrature sum of the differences in the azimuthal angle and pseudorapidity. The value of the isolation variable is required to be smaller than 6% for electrons and 15% for muons. Events with dilepton invariant mass $m_{\ell \ell } < 20\,\text {Ge}\text {V}$ ($\ell =\mathrm {e},\mathrm {\mu }$) are rejected to suppress backgrounds due to QCD multijet production and decays of low mass resonances. Additionally, leptons are required to be consistent with originating from the primary interaction vertex.

Jets are reconstructed from the PF candidates using the anti-$k_{\mathrm {T}}$ clustering algorithm with a distance parameter of 0.4 [57, 58]. The jet momentum is determined from the vectorial sum of all particle momenta in the jet, and is found from simulation to be within 5 to 10% of the true momentum over the relevant phase space of this analysis [59]. Pileup interactions can contribute additional tracks and calorimetric energy depositions to the jet momentum. To mitigate this effect, charged particles identified as originating from pileup vertices are discarded and an offset correction is applied to correct for remaining contributions [59]. The jet energy corrections are determined from measurements of the energy balance in dijet, multijet, photon+jet, and leptonically decaying $\mathrm {Z}$+jets events, and are applied as a function of the jet $p_{\mathrm {T}}$ and $\eta$ to both data and simulated events [59]. For this measurement, jets are selected if they fulfill the criteria $p_{\mathrm {T}} > 30\,\text {Ge}\text {V}$ and $|\eta |< 2.4$.

Jets originating from the hadronization of $\mathrm {b}$ quarks ($\mathrm {b}$ jets) are identified ($\mathrm {b}$ tagged) using the combined secondary vertex [60] algorithm, which combines lifetime information from tracks and secondary vertices. To achieve high purity, a working point is chosen such that the fraction of light-flavour jets with $p_{\mathrm {T}} > 30 \,\text {Ge}\text {V}$ that are falsely identified as $\mathrm {b}$ jets is $0.1\%$, resulting in an average efficiency of about 41% for genuine $\mathrm {b}$ jets and 2.2% for $\mathrm {c}$ jets [60].

In the same-flavour channels, $\mathrm {\mu ^+}\mathrm {\mu ^-}$ and $\mathrm {e}^+\mathrm {e}^-$, DY events are suppressed by excluding the region of the $\mathrm {Z}$ boson mass through the requirement $76< m_{\ell \ell } < 106\,\text {Ge}\text {V}$. In these channels, events are also required to contain at least one $\mathrm {b}$-tagged jet.

**Fig. 1:**
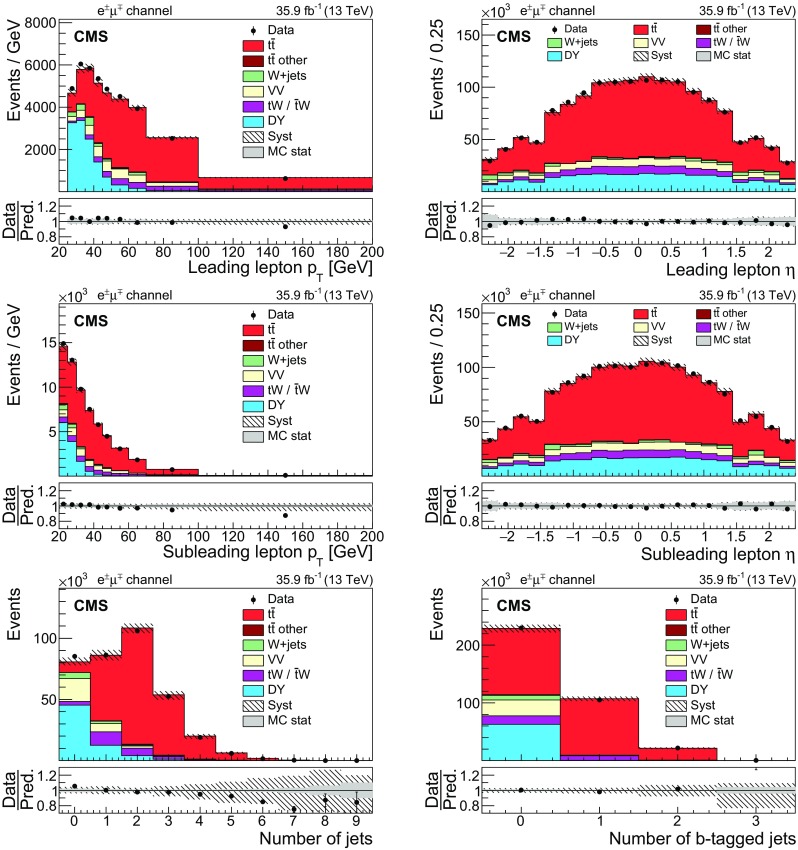
Distributions of the transverse momentum (left) and pseudorapidity (right) of the leading (upper) and subleading (middle) leptons in the $\mathrm {e}$ $^{\pm }$ $\mathrm {\mu }$ $^{{\mp }}$ channel after the event selection for the data (points) and the predictions for the signal and various backgrounds from the simulation (shaded histograms). The lower row shows the jet (left) and $\mathrm {b}$-tagged jet (right) multiplicity distributions. The vertical bars on the points represent the statistical uncertainties in the data. The hatched bands correspond to the systematic uncertainty in the ${\mathrm {t}\overline{\mathrm {t}}}$ signal MC simulation. The uncertainties in the integrated luminosity and background contributions are not included. The ratios of the data to the sum of the predicted yields are shown in the lower panel of each figure. Here, the solid gray band represents the contribution of the statistical uncertainty in the MC simulation

**Fig. 2:**
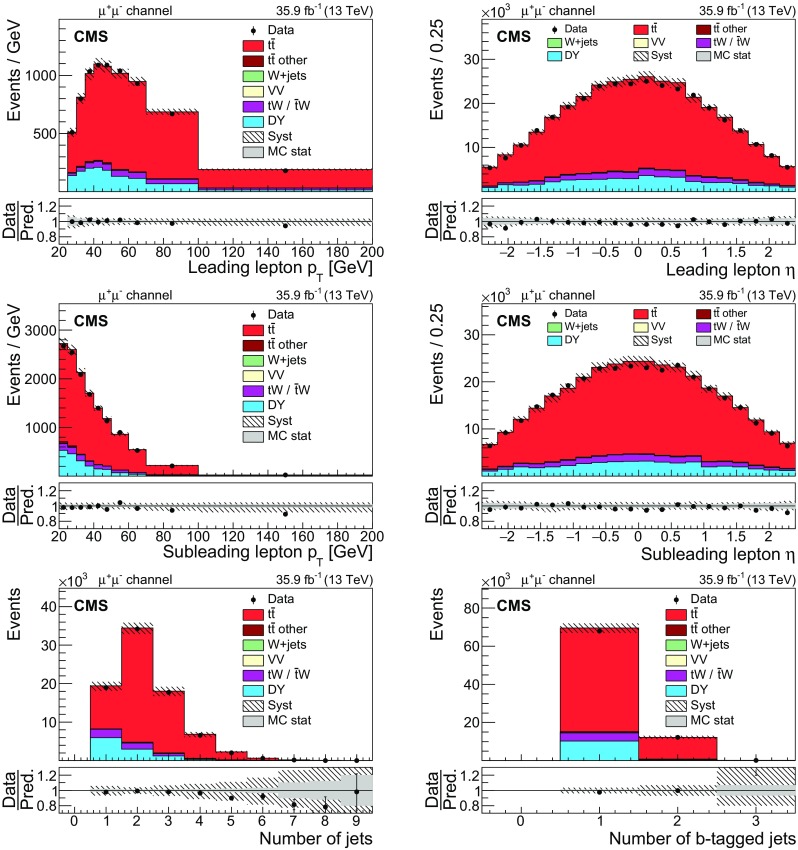
The same distributions as in Fig. 1, but for the $\mathrm {\mu ^+}\mathrm {\mu ^-}$ channel

**Fig. 3:**
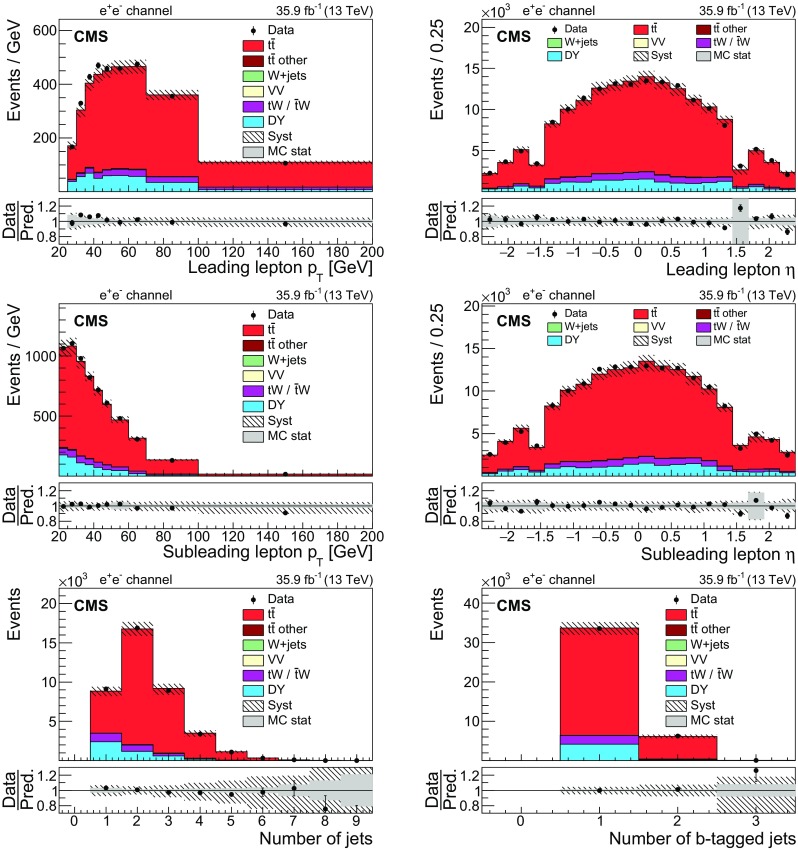
The same distributions as in Fig. 1, but for the $\mathrm {e}^+\mathrm {e}^-$ channel

Distributions of the leading and subleading lepton $p_{\mathrm {T}}$ and $\eta$, and the jet and $\mathrm {b}$-tagged jet multiplicities in events fulfilling the above selection criteria are shown in Figs. 1, 2 and 3 for the $\mathrm {e}$ $^{\pm }$ $\mathrm {\mu }$ $^{{\mp }}$, $\mathrm {\mu ^+}\mathrm {\mu ^-}$, and $\mathrm {e}^+\mathrm {e}^-$ channels, respectively. The event yields in the simulations are normalized to the corresponding cross section predictions, as explained in Sect. 2. Selected events include a very small contribution from ${\mathrm {t}\overline{\mathrm {t}}}$ processes in the lepton+jets decay channel (referred to as “${\mathrm {t}\overline{\mathrm {t}}}$ other” in the figures) in which one of the charged leptons originates from heavy-flavour hadron decay, misidentified hadrons, muons from light-meson decays, or electrons from unidentified photon conversions. Such leptons also lead to dilepton background in this analysis via $\mathrm {W}$+jets processes.

In all categories, the simulation is found to describe the data well within the systematic uncertainties, indicated by the bands in the figures.

## Event categories and fit procedure

The measurement is performed using a template fit to multidifferential distributions, divided into distinct event categories using the $\mathrm {b}$-tagged jet multiplicity, similar to the method utilized in a previous measurement [4]. In each of the same-flavour channels, two categories are defined, corresponding to events having 1 or 2 $\mathrm {b}$-tagged jets. Events with zero $\mathrm {b}$-tagged jets are not included since they are dominated by the DY background process. In the $\mathrm {e}$ $^{\pm }$ $\mathrm {\mu }$ $^{{\mp }}$ channel, three categories are defined, corresponding to events having 1, 2, or 0 or $\ge$ 3 $\mathrm {b}$-tagged jets. The templates describing the distributions for the signal and background events are taken from simulation. Categorizing the events by their $\mathrm {b}$-tagged jet multiplicity allows the efficiency $\epsilon _{\mathrm {b}}$ for selecting and identifying a $\mathrm {b}$ jet to be constrained. Previous measurements that used a template fit with dilepton events were restricted to the $\mathrm {e}$ $^{\pm }$ $\mathrm {\mu }$ $^{{\mp }}$ channel [1, 4]. In this analysis, the decay channels with two electrons and two muons are also included in the fit. In this way, additional constraints on the lepton identification efficiencies are obtained.

First, a visible ${\mathrm {t}\overline{\mathrm {t}}}$ cross section $\sigma _{{\mathrm {t}\overline{\mathrm {t}}}}^{\text {vis}}$, defined for a phase space corresponding to the experimentally accessible fiducial volume, as described in Sect. 6, is determined. For the visible cross section, the fit is used to constrain the systematic uncertainties from the data. Using the relation

1 $$\begin{aligned} \sigma _{\mathrm {t}\overline{\mathrm {t}}} = \frac{\sigma _{{\mathrm {t}\overline{\mathrm {t}}}}^{\text {vis}}}{A_{\ell \ell }}, \end{aligned}$$

the measured visible cross section is then extrapolated to the full phase space to obtain $\sigma _{\mathrm {t}\overline{\mathrm {t}}}$. Here, $A_{\ell \ell }$ denotes the acceptance, which is defined as the fraction of ${\mathrm {t}\overline{\mathrm {t}}}$ events that fulfill the selection criteria for the visible cross section. The acceptance incorporates the combined branching fraction for the $\mathrm {t}$ and $\overline{\mathrm {t}}$ quarks to decay to two charged leptons [29]. Apart from the free parameter of interest $\sigma _{{\mathrm {t}\overline{\mathrm {t}}}}^{\text {vis}}$, the parameters of the fit are the *J* nuisance parameters $\mathbf {\lambda } = (\lambda _1, \lambda _2, \ldots , \lambda _J)$ corresponding to the various sources of systematic uncertainty, discussed in detail in Sect. 5.

The likelihood function *L* is based on Poisson statistics:

2 $$\begin{aligned} L = \prod _{i} \frac{\mathrm {e}^{ -\nu _i } \nu _i^{n_i}}{n_i!}\, \prod _{j} \pi (\lambda _j), \end{aligned}$$

where *i* denotes the bin of the respective final-state distribution, and $\nu _i$ and $n_i$ are the expected and observed number of events in bin *i*, respectively. The symbol $\pi (\lambda _j)$ denotes a penalty term for the deviation of the nuisance parameter $\lambda _j$ from its nominal value according to its prior density distribution. A Gaussian prior density distribution is assumed for all nuisance parameters. The expectation values $\nu _i$ can be written as

3 $$\begin{aligned} \nu _i = s_i(\sigma _{{\mathrm {t}\overline{\mathrm {t}}}}^{\text {vis}},\mathbf {\lambda }) + \sum _{k} b_{k,i}^{\mathrm {MC}}(\mathbf {\lambda }). \end{aligned}$$

Here, $s_i$ denotes the expected number of ${\mathrm {t}\overline{\mathrm {t}}}$ signal events in bin *i* and the quantity $b_{k,i}^{\mathrm {MC}}$ represents the prediction of the number of background events in bin *i* from source *k*. The Minuit program [61] is used to minimize $-2 \ln {(L)}$ with *L* given in Eq. (2), and the Minos  [61] algorithm is used to estimate the uncertainties.

For the determination of the $\mathrm {b}$ tagging efficiencies, multinomial probabilities are used to describe the expected number of signal events with one $\mathrm {b}$-tagged jet, $s_{1\mathrm {b}}$, two $\mathrm {b}$-tagged jets, $s_{2\mathrm {b}}$, and zero or more than two $\mathrm {b}$-tagged jets, $s_{\text {other}}$:

4 $$\begin{aligned} s_{1\mathrm {b}}= & {} \mathcal {L} \sigma _{{\mathrm {t}\overline{\mathrm {t}}}}^{\text {vis}} \epsilon _{\ell \ell } 2 \epsilon _{\mathrm {b}}(1-C_{\mathrm {b}}\epsilon _{\mathrm {b}}), \end{aligned}$$

5 $$\begin{aligned} s_{2\mathrm {b}}= & {} {\mathcal {L} \sigma _{{\mathrm {t}\overline{\mathrm {t}}}}^{\text {vis}} \epsilon _{\ell \ell } \epsilon _{\mathrm {b}}^2 C_{\mathrm {b}} },\end{aligned}$$

6 $$\begin{aligned} s_{\text {other}}= & {} \mathcal {L} \sigma _{{\mathrm {t}\overline{\mathrm {t}}}}^{\text {vis}} \epsilon _{\ell \ell } (1-2\epsilon _{\mathrm {b}}(1-C_{\mathrm {b}} \epsilon _{\mathrm {b}})-\epsilon _{\mathrm {b}}^2C_{\mathrm {b}}), \end{aligned}$$

where $\mathcal {L}$ denotes the integrated luminosity and $\epsilon _{\ell \ell }$ is the efficiency for events in the visible phase space to pass the full selection described in Sect. 3. The quantity $C_{\mathrm {b}}$ corrects for any small correlations between the tagging of two $\mathrm {b}$ jets in an event, expressed as $C_{\mathrm {b}} = 4 s_{\text {all}} s_{2\mathrm {b}}/ (s_{1\mathrm {b}}+2 s_{2\mathrm {b}})^2$, where $s_{\text {all}}$ denotes the total number of signal events. The values for $\epsilon _{\ell \ell }$, $\epsilon _{\mathrm {b}}$, and $C_{\mathrm {b}}$ are directly determined from the ${\mathrm {t}\overline{\mathrm {t}}}$ signal simulation, expressing $\epsilon _{\mathrm {b}}$ as $(s_{1\mathrm {b}} + 2 s_{2\mathrm {b}})/2 s_{\text {all}}$. The values of these parameters for the nominal signal simulation in the $\mathrm {e}$ $^{\pm }$ $\mathrm {\mu }$ $^{{\mp }}$ channel are $\epsilon _{\mathrm {e}\mathrm {\mu }} = 0.49$, $\epsilon _{\mathrm {b}} = 0.30$, and $C_{\mathrm {b}} = 1.00$.

The overall selection efficiency $\epsilon _{\ell \ell }$ is a linear combination of the efficiencies $\epsilon _{\mathrm {e}\mathrm {\mu }}$, $\epsilon _{\mathrm {e}\mathrm {e}}$, and $\epsilon _{\mathrm {\mu }\mathrm {\mu }}$, in the three different dilepton channels, each given by the product of the two efficiencies for identifying a single lepton of the respective flavour. Prior to the fit, the muon identification uncertainty is smaller than that for electrons. By fitting the three dilepton decay channels simultaneously, the ratio of single-lepton efficiencies $\epsilon _\mathrm {e}$ and $\epsilon _\mathrm {\mu }$ is constrained. In the fit, the electron identification uncertainty is constrained to that for muons.

The values for $\epsilon _{\ell \ell }$, $\epsilon _{\mathrm {b}}$, $C_{\mathrm {b}}$, the number of signal events in each category, and the background rates depend on the nuisance parameters $\mathbf {\lambda }$. The dependence on the parameter $\lambda _j$ is modelled by a second-order polynomial that describes the quantity at the three values $\lambda _j=0,1,-1$, corresponding to the nominal value of the parameter and to a variation by +1 and $-\,1$ standard deviation, respectively. If a variation is only possible in one direction, a linear function is used to model the dependence on $\lambda _j$.

The events are further categorized by the number of additional non-$\mathrm {b}$-tagged jets in the event. Each of the seven previously described event categories is further divided by grouping together events with 0, 1, 2, or $\ge$ 3 additional non-$\mathrm {b}$-tagged jets, thus producing 28 disjoint event categories. For those categories that have events with at least one additional non-$\mathrm {b}$-tagged jet, the smallest $p_{\mathrm {T}}$ among those jets is used as the observable in the fit. For those categories containing events with zero additional non-$\mathrm {b}$-tagged jets, the total number of events in the category is used as the observable in the fit. The further division of events into these categories and the observable distributions from each category provide the sensitivity to constrain the modelling systematic uncertainties, such as those coming from variations in the scales for the matrix element (ME) and parton shower (PS) matching. For events with no additional jets, the total event yield is used.

The statistical uncertainty in the templates from simulation is taken into account by using pseudo-experiments. At each iteration, templates are varied within their statistical uncertainty. Templates created from different simulations are treated as statistically uncorrelated, while templates derived by varying weights in the simulation are treated as correlated. The template dependencies are rederived and the fit to data is repeated. Repeating this 30,000 times yields an approximately Gaussian distribution of the fitted value of the ${\mathrm {t}\overline{\mathrm {t}}}$ cross section (and of $m_\mathrm {\mathrm {t}} ^{\mathrm {MC}}$ in the combined fit) and of the vast majority of the nuisance parameters. The root-mean-square of each distribution is considered as an additional uncertainty from the event counts in the simulated samples for the corresponding nuisance parameter.

**Fig. 4:**
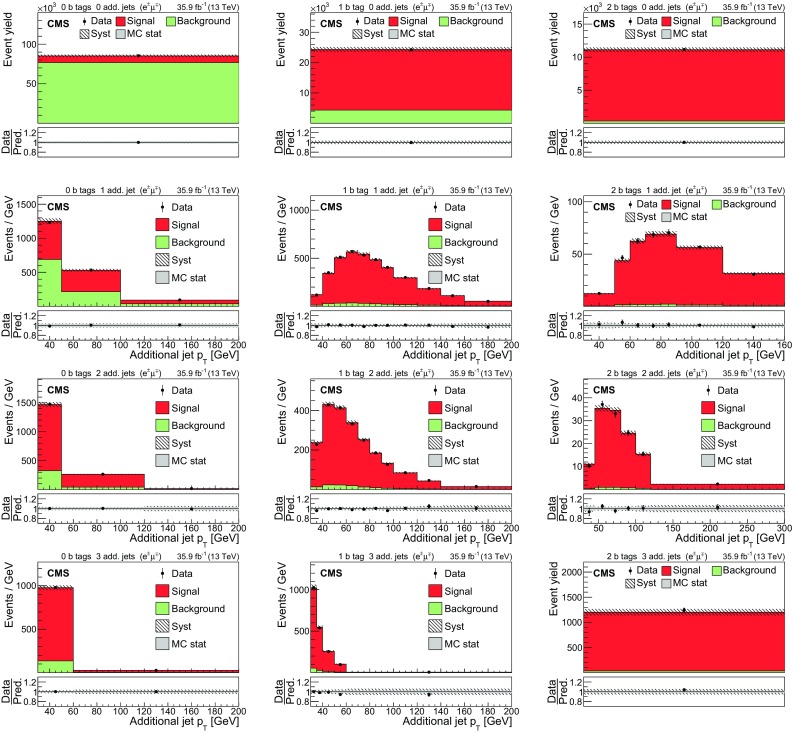
Distributions in the $\mathrm {e}$ $^{\pm }$ $\mathrm {\mu }$ $^{{\mp }}$ channel after the fit to the data. In the left column events with zero or three or more $\mathrm {b}$-tagged jets are shown. The middle (right) column shows events with exactly one (two) $\mathrm {b}$-tagged jets. Events with zero, one, two, or three or more additional non-$\mathrm {b}$-tagged jets are shown in the first, second, third, and fourth row, respectively. The hatched bands correspond to the total uncertainty in the sum of the predicted yields including all correlations. The ratios of the data to the sum of the simulated yields after the fit are shown in the lower panel of each figure. Here, the solid gray band represents the contribution of the statistical uncertainty in the MC simulation

**Fig. 5:**
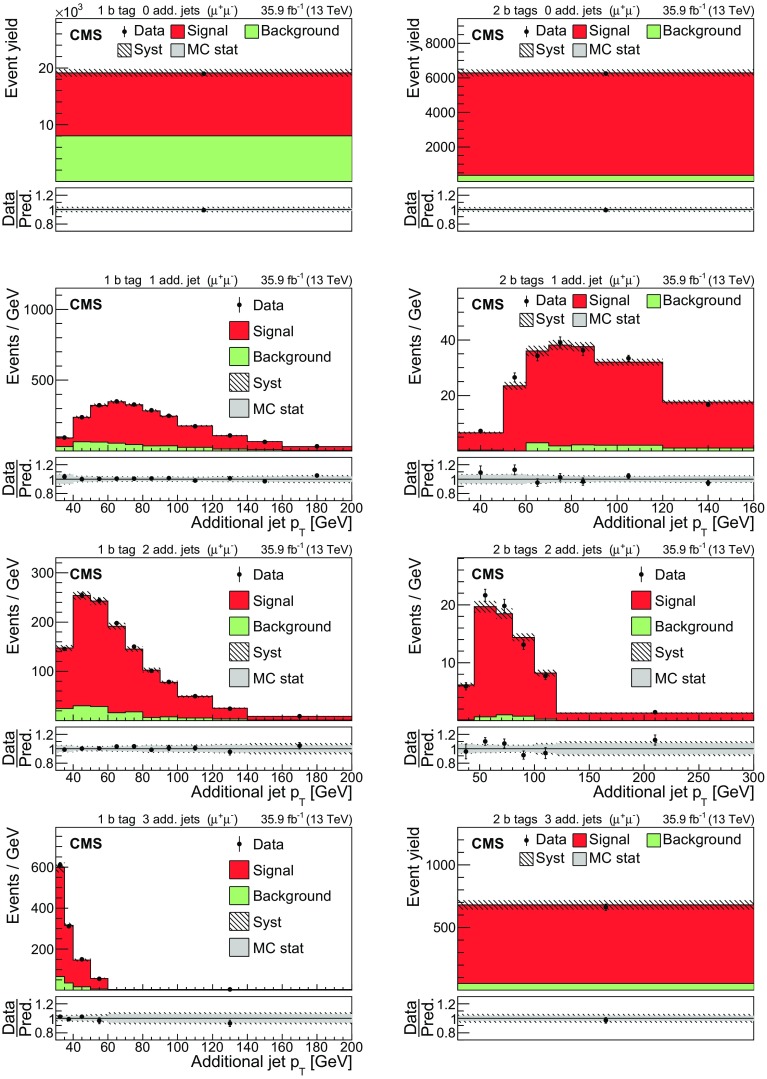
Distributions in the $\mathrm {\mu ^+}\mathrm {\mu ^-}$ channel after the fit to the data. The left (right) column shows events with exactly one (two) $\mathrm {b}$-tagged jets. Events with zero, one, two, or three or more additional non-$\mathrm {b}$-tagged jets are shown in the first, second, third, and fourth row, respectively. The hatched bands correspond to the total uncertainty in the sum of the predicted yields including all correlations. The ratios of the data to the sum of the simulated yields after the fit are shown in the lower panel of each figure. Here, the solid gray band represents the contribution of the statistical uncertainty in the MC simulation

**Fig. 6:**
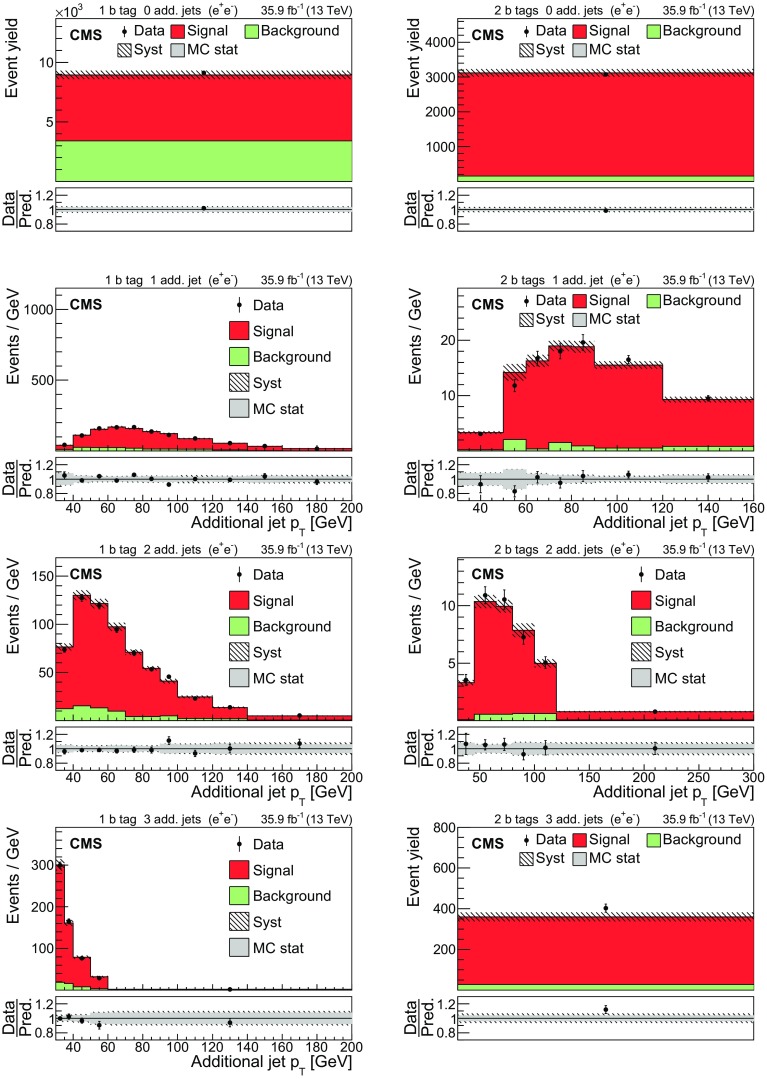
Same distributions as in Fig. 5, but in the $\mathrm {e}^+\mathrm {e}^-$ channel

The input distributions to the fit are shown in Figs. 4, 5 and 6, where the data are compared to the signal and background distributions resulting from the fit to the data. In the top row, the number of events without additional non-$\mathrm {b}$-tagged jets is displayed. For events with at least one additional non-$\mathrm {b}$-tagged jet, the $p_{\mathrm {T}}$ distributions of the non-$\mathrm {b}$-tagged jet with the smallest $p_{\mathrm {T}}$ in the respective category is considered, except for the category corresponding to events with 2 $\mathrm {b}$-tagged jets and at least three additional non-$\mathrm {b}$-tagged jets, where the statistical uncertainty of the simulation is high. This distribution is chosen in order to constrain the jet energy scale at lower jet $p_{\mathrm {T}}$, where the corresponding systematic uncertainty is larger [59]. Good agreement is found between the data and the simulation.

## Systematic uncertainties

The contributions from each source of systematic uncertainty are represented by nuisance parameters (see Sect. 4). For each uncertainty, the simulation is used to construct template histograms that describe the expected signal and background distributions for a given nuisance parameter variation. In the fit of the templates to the data, the best values for $\sigma _{{\mathrm {t}\overline{\mathrm {t}}}}^{\text {vis}}$ (and $m_\mathrm {\mathrm {t}} ^{\mathrm {MC}}$ in the case of the combined fit) and all nuisance parameters are determined, as described in Sect. 4. The prior probability density functions for the nuisance parameters have a Gaussian shape. Table 1 shows the value of the contributions of the uncertainties after the fit.

**Table 1:** The relative uncertainties in $\sigma _{{\mathrm {t}\overline{\mathrm {t}}}}^{\text {vis}}$ and $\sigma _{\mathrm {t}\overline{\mathrm {t}}}$ and their sources, as obtained from the template fit. The uncertainty in the integrated luminosity and the MC statistical uncertainty are determined separately. The individual uncertainties are given without their correlations, which are however accounted for in the total uncertainties. Extrapolation uncertainties only affect $\sigma _{\mathrm {t}\overline{\mathrm {t}}}$. For these uncertainties, the ± notation is used if a positive variation produces an increase in $\sigma _{\mathrm {t}\overline{\mathrm {t}}}$, while the ${\mp }$ notation is used otherwise

Source	Uncertainty (%)
Trigger	0.3
Lepton ident./isolation	2.0
Muon momentum scale	0.1
Electron momentum scale	0.1
Jet energy scale	0.4
Jet energy resolution	0.4
$\mathrm {b}$ tagging	0.4
Pileup	0.1
${\mathrm {t}\overline{\mathrm {t}}}$ ME scale	0.2
$\mathrm {t}\mathrm {W}$ ME scale	0.2
DY ME scale	0.1
PDF	1.1
Top quark $p_{\mathrm {T}}$	0.5
ME/PS matching	0.2
UE tune	0.3
${\mathrm {t}\overline{\mathrm {t}}}$ ISR scale	0.4
$\mathrm {t}\mathrm {W}$ ISR scale	0.1
${\mathrm {t}\overline{\mathrm {t}}}$ FSR scale	0.8
$\mathrm {t}\mathrm {W}$ FSR scale	0.1
$\mathrm {b}$ quark fragmentation	0.7
$\mathrm {b}$ hadron BF	0.1
Colour reconnection	0.3
DY background	0.9
$\mathrm {t}\mathrm {W}$ background	1.1
Diboson background	0.2
$\mathrm {W}$+jets background	0.2
${\mathrm {t}\overline{\mathrm {t}}}$ background	0.2
Statistical	0.2
Integrated luminosity	2.5
MC statistical	1.1
Total $\sigma _{{\mathrm {t}\overline{\mathrm {t}}}}^{\text {vis}}$ uncertainty	3.8
Extrapolation uncertainties	
${\mathrm {t}\overline{\mathrm {t}}}$ ME scale	${\mp }^{0.3}_{0.1}$
PDF	$\pm ^{0.8}_{0.6}$
Top quark $p_{\mathrm {T}}$	${\mp }^{0.5}_{<0.1}$
${\mathrm {t}\overline{\mathrm {t}}}$ ISR scale	${\mp }^{0.1}_{<0.1}$
${\mathrm {t}\overline{\mathrm {t}}}$ FSR scale	$\pm ^{0.1}_{<0.1}$
UE tune	<0.1
Total $\sigma _{\mathrm {t}\overline{\mathrm {t}}}$ uncertainty	4.0

Most of the experimental uncertainties are determined from ancillary measurements in which data and simulation are compared and small corrections to the simulation, referred to as scale factors (SFs), are determined. To assess the impact of the uncertainty in these corrections, the SFs are varied within their uncertainty and the analysis is repeated.

The trigger efficiencies are determined using multiple independent methods, which show agreement within 0.3%. An additional statistical uncertainty arises because the SFs are determined from the data in intervals of $p_{\mathrm {T}}$ and $\eta$.

The uncertainty in the SFs of the lepton identification efficiency is typically 1.5% for electrons and 1.2% for muons, with a small dependency on the lepton $p_{\mathrm {T}}$ and $\eta$. The uncertainties in the calibration of the muon and electron momentum scales are included as nuisance parameters for each lepton separately. Their impact on the measurement is negligible.

The impact of the jet energy scale (JES) uncertainties is estimated by varying the jet momenta within the JES uncertainties, split into 18 contributions [59]. To account for the jet energy resolution (JER), the SFs are varied within their $|\eta |$-dependent uncertainties [62].

The uncertainties associated with the $\mathrm {b}$ tagging efficiency are determined by varying the related corrections for the simulation of $\mathrm {b}$ jets and light-flavour jets, split into 16 orthogonal contributions for $\mathrm {b}$ jets. These uncertainties depend on the $p_{\mathrm {T}}$ of each jet and amount to approximately 1.5% for $\mathrm {b}$ jets in ${\mathrm {t}\overline{\mathrm {t}}}$ signal events [60].

The uncertainty in the modelling of the number of pileup events is obtained by changing the inelastic pp cross section, which is used to model the pileup in simulation, by ±4.6% [48].

The integrated luminosity uncertainty is not included in the fit as a nuisance parameter, but treated as an external uncertainty. It is estimated to be 2.5% [63].

The ME scale uncertainties for the simulation of the ${\mathrm {t}\overline{\mathrm {t}}}$ and DY are assessed by varying the renormalization and factorization scale choices in powheg by factors of two up and down independently [64, 65], avoiding cases where $\mu _\mathrm {f}/\mu _\mathrm {r} = 1/4$ or 4.

To estimate the uncertainty due to the NLO generator, the powheg ${\mathrm {t}\overline{\mathrm {t}}}$ signal sample is replaced by a ${\mathrm {t}\overline{\mathrm {t}}}$ sample generated using the MadGraph 5_amc@nlo program with FxFx matching [66]. This uncertainty is only included in the combined measurement of $\sigma _{\mathrm {t}\overline{\mathrm {t}}}$ and $m_\mathrm {\mathrm {t}} ^{\mathrm {MC}}$ (Sect. 7) in order to compare with the latest direct top quark mass measurement from CMS in the lepton+jets channel [31].

The PDF uncertainty is estimated using the 28 orthogonal Hessian eigenvectors of the CT14 [53] PDF, which are used as independent inputs to the fit.

Differential measurements of $\sigma _{\mathrm {t}\overline{\mathrm {t}}}$ at $\sqrt{s} = 13 \,\text {Te}\text {V}$ have demonstrated that the $p_{\mathrm {T}}$ distribution of the top quark is softer than predicted by the powheg simulation [67–69]. An additional uncertainty, referred to as “Top quark $p_{\mathrm {T}}$ ”, is estimated by reweighting the simulation. This nuisance parameter has a one-sided prior distribution.

The uncertainty due to the matching of the ME to the PS in simulation is estimated by varying the $h_{\text {damp}}$ parameter in powheg, as described in Ref. [40]. The uncertainty due to the assumptions in the UE tune is estimated by varying the tuning parameters [40]. The impact of the PS scale uncertainty is estimated by varying the initial-state radiation (ISR) and the final-state radiation (FSR) scales by a factor of two up and down [41], similar to the case of renormalization and factorization scales.

The uncertainties due to the assumed $\mathrm {b}$ hadron branching fraction (BF) and fragmentation are taken into account following the procedures described in Ref. [31]. For the fragmentation, variations of the Bowler–Lund fragmentation function [70] and the comparison to the Peterson fragmentation function [71] are considered.

The effects of colour reconnection (CR) processes on the top quark final state are estimated by enabling early resonance decays (ERD) in pythia. In the nominal sample, ERD are turned off. Alternative colour reconnection models are considered, such as “gluon move” [72] and “QCD inspired” [73], since they were found to potentially have relevant effects for the measurement of the top quark mass [31].

For the uncertainties related to the background contributions, prior normalization uncertainties of 30% are assumed [74]. The contributions of these uncertainties are small and/or strongly constrained in the fit. For the DY background, separate nuisance parameters are used for each $\mathrm {b}$-tagged jet category in order to remove the dependence of the fit result on the prediction of the $\mathrm {b}$-tagged jet multiplicity distribution by the DY MC simulation. Similarly, the DY background is given an additional uncertainty of 5, 10, 30, and 50% for events with exactly 0, 1, 2, and 3 or more jets, respectively. The first three numbers are estimated by performing scale variations in $\mathrm {W}$+jets predictions with NLO precision, whereas the last one is assigned conservatively.

**Fig. 7:**
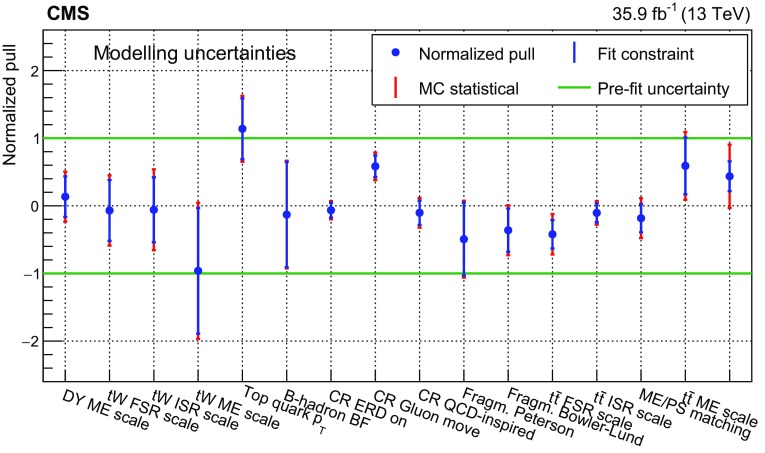
Normalized pulls and constraints of the nuisance parameters related to the modelling uncertainties for the cross section fit. The markers denote the fitted values, while the inner vertical bars represent the constraint and the outer vertical bars denote the additional uncertainty as determined from pseudo-experiments. The constraint is defined as the ratio of the post-fit uncertainty to the pre-fit uncertainty of a given nuisance parameter, while the normalized pull is the difference between the post-fit and the pre-fit values of the nuisance parameter normalized to its pre-fit uncertainty. The horizontal lines at $\pm \,1$ represent the pre-fit uncertainty

In total, 103 uncertainty sources are used in the fit. In Fig. 7, the normalized pulls and constraints for the nuisance parameters related to the modelling uncertainties are shown. For each nuisance parameter, the normalized pull is defined as the difference between the best-fit and the input values, normalized to the pre-fit uncertainty, and the constraint is defined as the ratio of the post-fit to the pre-fit uncertainty. The vast majority of the nuisance parameters lie within one standard deviation of their priors, reflecting the good agreement of the nominal simulation with the data. Most ${\mathrm {t}\overline{\mathrm {t}}}$ signal uncertainties show significant constraints with respect to their prior uncertainty, illustrating the strength of the analysis ansatz. The nuisance parameter for the $p_{\mathrm {T}}$ distribution of the top quarks is pulled by one standard deviation. This is expected since it is known that the observed $p_{\mathrm {T}}$ distribution of the top quark is softer than predicted by the simulation [68, 69].

## Cross section measurement

The visible cross section is defined for ${\mathrm {t}\overline{\mathrm {t}}}$ events in the fiducial region with two oppositely charged leptons (electron or muon). Contributions from leptonically decaying $\mathrm {\tau }$ leptons are included. The leading lepton is required to have $p_{\mathrm {T}} > 25\,\text {Ge}\text {V}$, and the subleading lepton must have $p_{\mathrm {T}} >20\,\text {Ge}\text {V}$. Both leptons have to be in the range $|\eta | < 2.4$. From the likelihood fit, described in Sect. 4, the visible cross section is measured to be

$$\begin{aligned} \sigma _{{\mathrm {t}\overline{\mathrm {t}}}}^{\text {vis}}= & {} 25.61 \pm 0.05 \,\text {(stat)} \pm 0.75 \,\text {(syst)} \pm 0.64 \,\text {(lumi)} \,\text {pb} . \end{aligned}$$

Here, the uncertainties denote the statistical uncertainty, the systematic uncertainty, and that coming from the uncertainty in the integrated luminosity. The full list of uncertainties is presented in Table 1.

The total cross section $\sigma _{\mathrm {t}\overline{\mathrm {t}}}$ is obtained by extrapolating the measured visible cross section to the full phase space. As explained in Sect. 4, the extrapolation is described by a multiplicative acceptance correction factor $A_{\ell \ell }$ (see Eq. (1)). The extrapolation uncertainty is determined for each relevant model systematic source *j* as described in the following: all nuisance parameters except the one under study are fixed to their post-fit values; the nuisance parameter $\lambda _j$ is set to values $+1$ and $-\,1$, and the variations of $A_{\ell \ell }$ are recorded. The resulting variations of $\sigma _{\mathrm {t}\overline{\mathrm {t}}}$ with respect to the nominal value, obtained with the post-fit value of $\lambda _j$, are taken as the additional extrapolation uncertainties. The individual uncertainties in $\sigma _{\mathrm {t}\overline{\mathrm {t}}}$ from these sources are summed in quadrature to estimate the total systematic uncertainty, as summarized in Table 1. A fixed value of $m_\mathrm {\mathrm {t}} ^{\mathrm {MC}} = 172.5 \,\text {Ge}\text {V}$ is chosen in the simulation, and no uncertainty is assigned.

The total cross section $\sigma _{\mathrm {t}\overline{\mathrm {t}}}$ is measured to be

$$\begin{aligned} \sigma _{{\mathrm {t}\overline{\mathrm {t}}}}= & {} 803 \pm 2 \,\text {(stat)} \pm 25 \,\text {(syst)} \pm 20 \,\text {(lumi)} \,\text {pb} . \end{aligned}$$

As shown in Table 1, in comparison to the fiducial cross section, the relative systematic uncertainty in the total cross section is marginally increased. The result is in good agreement with the theoretical calculation at NNLO+NNLL, which predicts a ${\mathrm {t}\overline{\mathrm {t}}}$ cross section of $832~^{+20}_{-29}\, (\text {scale}) \pm 35 ~(\text {PDF}+\alpha _S ) \,\text {pb}$, as described in Sect. 2.

An independent cross section measurement is performed using a simple event-counting method and a more restrictive event selection, following closely the analysis of Ref. [75]. The analysis uses events in the $\mathrm {e}$ $^{\pm }$ $\mathrm {\mu }$ $^{{\mp }}$ channel with at least two jets, at least one of which is $\mathrm {b}$ tagged. The cross section is measured to be $\sigma _{\mathrm {t}\overline{\mathrm {t}}} = 804 \pm 2 \,\text {(stat)} \pm 31 \,\text {(syst)} \pm 20 \,\text {(lumi)} \,\text {pb}$, in good agreement with the main result.

## Simultaneous measurement of $\sigma _{\mathrm {t}\overline{\mathrm {t}}}$ and $m_\mathrm {\mathrm {t}} ^{\mathrm {MC}}$

The analysis is designed such that the dependence of the measured ${\mathrm {t}\overline{\mathrm {t}}}$ cross section on $m_\mathrm {\mathrm {t}} ^{\mathrm {MC}}$ is small. However, because of the impact of the top quark mass on the simulated detector efficiency and acceptance, the measurement is expected to have a residual dependence on the chosen value of $m_\mathrm {\mathrm {t}} ^{\mathrm {MC}}$. In previous measurements, this dependence was determined by repeating the analysis with varied mass values.

Here, the approach proposed in Refs. [5, 30] is followed. The value of $m_\mathrm {\mathrm {t}} ^{\mathrm {MC}}$ is introduced in the fit as an additional free parameter. In the simultaneous fit, $\sigma _{\mathrm {t}\overline{\mathrm {t}}}$ and $m_\mathrm {\mathrm {t}} ^{\mathrm {MC}}$ are directly constrained from the data. The resulting $\sigma _{\mathrm {t}\overline{\mathrm {t}}}$ and its uncertainty therefore account for the dependence on $m_\mathrm {\mathrm {t}} ^{\mathrm {MC}}$ and can be used, e.g. for the extraction of $m_\mathrm {\mathrm {t}}$ and $\alpha _S$ using fixed-order calculations. The value of $m_\mathrm {\mathrm {t}} ^{\mathrm {MC}}$, in turn, can be compared to the results of direct measurements using, e.g. kinematic fits [31].

**Fig. 8:**
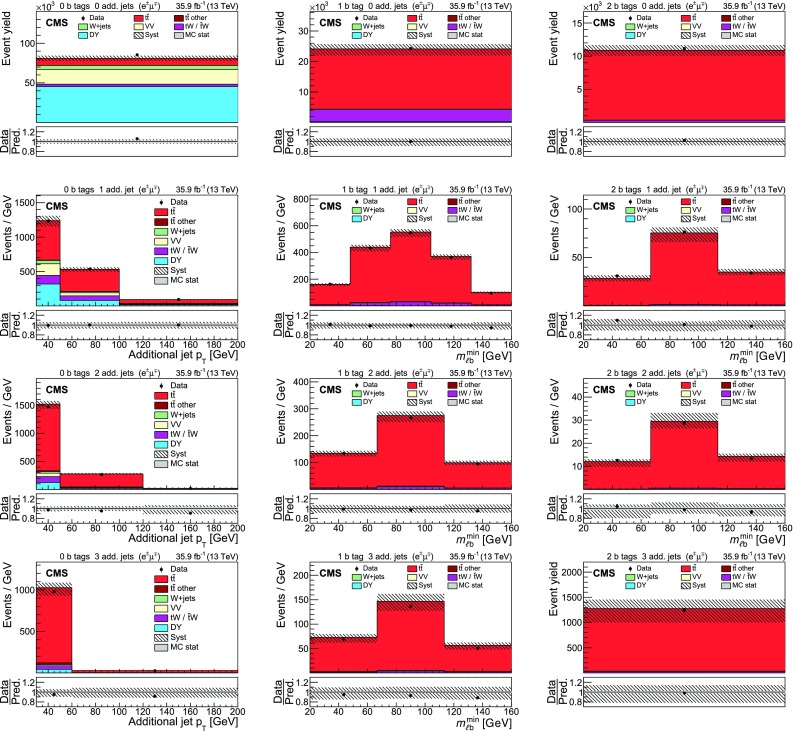
Comparison of data (points) and pre-fit distributions of the expected signal and backgrounds from simulation (shaded histograms) used in the simultaneous fit of $\sigma _{\mathrm {t}\overline{\mathrm {t}}}$ and $m_\mathrm {\mathrm {t}} ^{\mathrm {MC}}$ in the $\mathrm {e}$ $^{\pm }$ $\mathrm {\mu }$ $^{{\mp }}$ channel. In the left column events with zero or three or more $\mathrm {b}$-tagged jets are shown. The middle (right) column shows events with exactly one (two) $\mathrm {b}$-tagged jets. Events with zero, one, two, or three or more additional non-$\mathrm {b}$-tagged jets are shown in the first, second, third, and fourth row, respectively. The hatched bands correspond to the total uncertainty in the sum of the predicted yields. The ratios of data to the sum of the predicted yields are shown in the lower panel of each figure. Here, the solid gray band represents the contribution of the statistical uncertainty

In contrast to the $\sigma _{\mathrm {t}\overline{\mathrm {t}}}$ measurement presented in Sect. 6, the sensitivity of the simultaneous fit to $m_\mathrm {\mathrm {t}} ^{\mathrm {MC}}$ is maximized by introducing a new observable: the minimum invariant mass $m_{\ell \mathrm {b}}^{\text {min}}$, which is defined as the smallest invariant mass found when combining the charged leptons with the $\mathrm {b}$ jets in an event. To minimize the impact from background, only the $\mathrm {e}$ $^{\pm }$ $\mathrm {\mu }$ $^{{\mp }}$ sample is used. The simultaneous fit of $\sigma _{\mathrm {t}\overline{\mathrm {t}}}$ and $m_\mathrm {\mathrm {t}} ^{\mathrm {MC}}$ is performed in 12 mutually exclusive categories, according to the number of $\mathrm {b}$-tagged jets and of additional non-$\mathrm {b}$-tagged jets in the event. The same observables as in Fig. 4 are used as input to the fit, where the jet $p_{\mathrm {T}}$ spectrum is replaced by the $m_{\ell \mathrm {b}}^{\text {min}}$ distribution in categories with at least one $\mathrm {b}$-tagged jet, as shown in Fig. 8.

To construct the templates describing the dependence of the final-state distributions on $m_\mathrm {\mathrm {t}} ^{\mathrm {MC}}$, separate MC simulation samples of ${\mathrm {t}\overline{\mathrm {t}}}$ and $\mathrm {t}\mathrm {W}$ production are used in which $m_\mathrm {\mathrm {t}} ^{\mathrm {MC}}$ is varied in the range $m_\mathrm {\mathrm {t}} ^{\mathrm {MC}} = 172.5 \pm 3 \,\text {Ge}\text {V}$. The data and MC samples, the event selection, the modelling of the systematic uncertainties, and the fit procedure are identical to those described in Sect. 4. In the simultaneous fit, the same systematic uncertainties are included as in a previous CMS measurement [31] of the $m_\mathrm {\mathrm {t}} ^{\mathrm {MC}}$. The results of the two measurements are thus directly comparable.

**Fig. 9:**
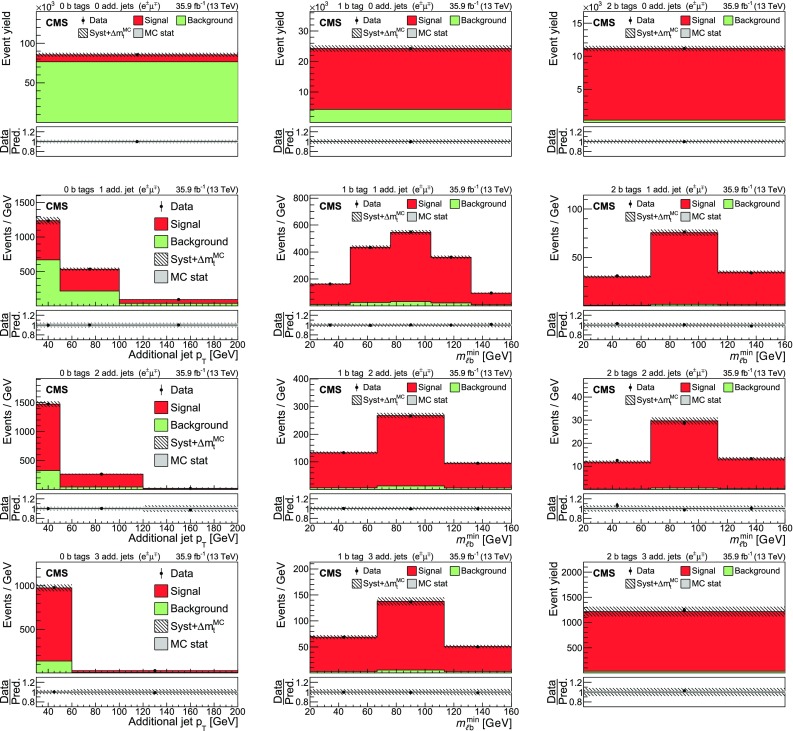
Comparison of data (points) and post-fit distributions of the expected signal and backgrounds from simulation (shaded histograms) used in the simultaneous fit of $\sigma _{\mathrm {t}\overline{\mathrm {t}}}$ and $m_\mathrm {\mathrm {t}} ^{\mathrm {MC}}$ in the $\mathrm {e}$ $^{\pm }$ $\mathrm {\mu }$ $^{{\mp }}$ channel. In the left column events with zero or three or more $\mathrm {b}$-tagged jets are shown. The middle (right) column shows events with exactly one (two) $\mathrm {b}$-tagged jets. Events with zero, one, two, or three or more additional non-$\mathrm {b}$-tagged jets are shown in the first, second, third, and fourth row, respectively. The hatched bands correspond to the total uncertainty in the sum of the predicted yields and include the contribution from the top quark mass ($\varDelta m_\mathrm {\mathrm {t}} ^{\mathrm {MC}}$). The ratios of data to the sum of the predicted yields are shown in the lower panel of each figure. Here, the solid gray band represents the contribution of the statistical uncertainty

Comparisons of the data and the prediction from the MC simulation before and after the fit are presented in Figs. 8 and 9, respectively. Good agreement is found in both cases.

The result of the fit is found to be stable against the choice of the fit distributions, and the introduction of the $m_{\ell \mathrm {b}}^{\text {min}}$ distribution was confirmed not to alter the final result on $\sigma _{\mathrm {t}\overline{\mathrm {t}}}$ or the behaviour with respect to the nuisance parameters. The procedure is calibrated by performing fits where data is replaced by simulations with different $m_\mathrm {\mathrm {t}} ^{\mathrm {MC}}$ hypotheses: full closure of the method is obtained and no additional correction is applied. The effect of the statistical uncertainty in the simulation on the fit results is estimated as explained in Sect. 4 and is considered as an additional uncertainty. The results for $\sigma _{\mathrm {t}\overline{\mathrm {t}}}$ and $m_\mathrm {\mathrm {t}} ^{\mathrm {MC}}$ are

$$\begin{aligned} \sigma _{\mathrm {t}\overline{\mathrm {t}}}&= 815 \pm 2 \,\text {(stat)} \pm 29 \,\text {(syst)} \pm 20 \,\text {(lumi)} \,\text {pb} , \\ m_\mathrm {\mathrm {t}} ^{\mathrm {MC}}&= 172.33 \pm 0.14 \,\text {(stat)} \,^{+0.66}_{-0.72} \,\text {(syst)} \,\text {Ge}\text {V} . \end{aligned}$$

The value for the cross section is in good agreement with the result obtained for a fixed value of $m_\mathrm {\mathrm {t}} ^{\mathrm {MC}} = 172.5 \,\text {Ge}\text {V}$, reported in Sect. 6. The correlation between the two parameters is found to be $12\%$.

**Fig. 10:**
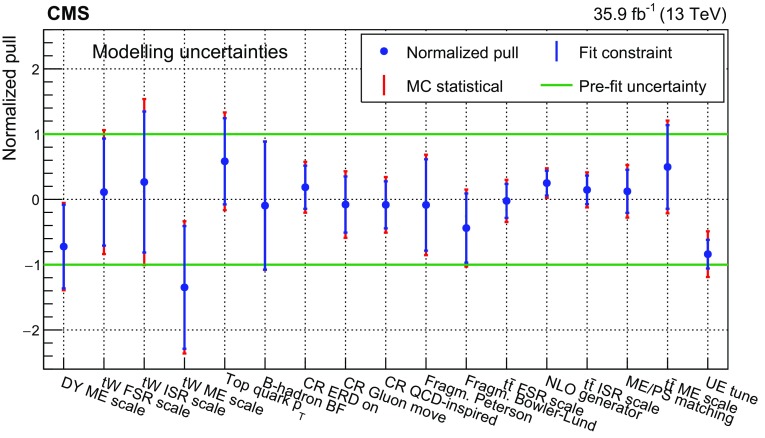
Normalized pulls and constraints of the nuisance parameters related to the modelling uncertainties for the simultaneous fit of $\sigma _{\mathrm {t}\overline{\mathrm {t}}}$ and $m_\mathrm {\mathrm {t}} ^{\mathrm {MC}}$. The markers denote the fitted value, while the inner vertical bars represent the constraint and the outer vertical bars denote the additional uncertainty as determined from pseudo-experiments. The constraint is defined as the ratio of the post-fit uncertainty to the pre-fit uncertainty of a given nuisance parameter, while the normalized pull is the difference between the post-fit and the pre-fit values of the nuisance parameter normalized to its pre-fit uncertainty. The horizontal lines at $\pm 1$ represent the pre-fit uncertainty

The results of the simultaneous fit to $\sigma _{\mathrm {t}\overline{\mathrm {t}}}$ and $m_\mathrm {\mathrm {t}} ^{\mathrm {MC}}$ are summarized in Tables 2 and 3, respectively, together with the contribution of each systematic uncertainty to the total uncertainty. Normalized pulls and constraints of the nuisance parameters related to modelling uncertainties are shown in Fig. 10. The nuisance parameters displayed in this figure show similar trends to those in Fig. 7, described above. Here, the constraints on the nuisance parameters tend to be less stringent because only data in the $\mathrm {e}$ $^{\pm }$ $\mathrm {\mu }$ $^{{\mp }}$ channel are used to determine the two parameters of interest, using mostly the $m_{\ell \mathrm {b}}^{\text {min}}$ spectra in place of the jet $p_{\mathrm {T}}$ distributions within the jet and $\mathrm {b}$-tagged jet categories.

As a cross-check, a measurement of $m_\mathrm {\mathrm {t}} ^{\mathrm {MC}}$ is performed by fitting a single $m_{\ell \mathrm {b}}^{\text {min}}$ distribution containing all events with at least one $\mathrm {b}$-tagged jet. The resulting value is $m_\mathrm {\mathrm {t}} ^{\mathrm {MC}}$ = $171.92 \pm 0.13 \,\text {(stat)} \,^{+0.76}_{-0.77} \,\text {(syst)} \,\text {Ge}\text {V}$. Since the uncorrelated uncertainty with respect to the main result is estimated to be at least $0.54 \,\text {Ge}\text {V}$, which is larger than the difference between the two measurements, the two results are in good agreement.

**Table 2:** The same as Table 1, but for the simultaneous fit of $\sigma _{\mathrm {t}\overline{\mathrm {t}}}$ and $m_\mathrm {\mathrm {t}} ^{\mathrm {MC}}$

Source	Uncertainty (%)
Trigger	0.4
Lepton ident./isolation	2.2
Muon momentum scale	0.2
Electron momentum scale	0.2
Jet energy scale	0.7
Jet energy resolution	0.5
$\mathrm {b}$ tagging	0.3
Pileup	0.3
${\mathrm {t}\overline{\mathrm {t}}}$ ME scale	0.5
$\mathrm {t}\mathrm {W}$ ME scale	0.7
DY ME scale	0.2
NLO generator	1.2
PDF	1.1
$m_\mathrm {\mathrm {t}} ^{\mathrm {MC}}$	0.4
Top quark $p_{\mathrm {T}}$	0.5
ME/PS matching	0.2
UE tune	0.3
${\mathrm {t}\overline{\mathrm {t}}}$ ISR scale	0.4
$\mathrm {t}\mathrm {W}$ ISR scale	0.4
${\mathrm {t}\overline{\mathrm {t}}}$ FSR scale	1.1
$\mathrm {t}\mathrm {W}$ FSR scale	0.2
$\mathrm {b}$ quark fragmentation	1.0
$\mathrm {b}$ hadron BF	0.2
Colour reconnection	0.4
DY background	0.8
$\mathrm {t}\mathrm {W}$ background	1.1
Diboson background	0.3
$\mathrm {W}$+jets background	0.3
${\mathrm {t}\overline{\mathrm {t}}}$ background	0.2
Statistical	0.2
Integrated luminosity	2.5
MC statistical	1.2
Total $\sigma _{{\mathrm {t}\overline{\mathrm {t}}}}^{\text {vis}}$ uncertainty	4.2
Extrapolation uncertainties	
${\mathrm {t}\overline{\mathrm {t}}}$ ME scale	${\mp }^{0.4}_{<0.1}$
PDF	$\pm ^{0.8}_{0.6}$
Top quark $p_{\mathrm {T}}$	$\pm ^{0.2}_{0.3}$
${\mathrm {t}\overline{\mathrm {t}}}$ ISR scale	${\mp }^{0.2}_{<0.1}$
${\mathrm {t}\overline{\mathrm {t}}}$ FSR scale	±0.1
UE tune	<0.1
$m_\mathrm {\mathrm {t}} ^{\mathrm {MC}}$	${\mp }^{0.2}_{0.3}$
Total $\sigma _{\mathrm {t}\overline{\mathrm {t}}}$ uncertainty	$^{+4.3}_{-4.2}$

**Table 3:** The absolute uncertainties in $m_\mathrm {\mathrm {t}} ^{\mathrm {MC}}$ and their sources, from the simultaneous fit of $\sigma _{\mathrm {t}\overline{\mathrm {t}}}$ and $m_\mathrm {\mathrm {t}} ^{\mathrm {MC}}$. The MC statistical uncertainty is determined separately. The individual uncertainties are given without their correlations, which are however accounted for in the total uncertainties

Source	Uncertainty ($\text {Ge}\text {V}$)
Trigger	0.02
Lepton ident./isolation	0.02
Muon momentum scale	0.03
Electron momentum scale	0.10
Jet energy scale	0.57
Jet energy resolution	0.09
$\mathrm {b}$ tagging	0.12
Pileup	0.09
${\mathrm {t}\overline{\mathrm {t}}}$ ME scale	0.18
$\mathrm {t}\mathrm {W}$ ME scale	0.02
DY ME scale	0.06
NLO generator	0.14
PDF	0.05
$\sigma _{{\mathrm {t}\overline{\mathrm {t}}}}$	0.09
Top quark $p_{\mathrm {T}}$	0.04
ME/PS matching	0.16
UE tune	0.03
${\mathrm {t}\overline{\mathrm {t}}}$ ISR scale	0.16
$\mathrm {t}\mathrm {W}$ ISR scale	0.02
${\mathrm {t}\overline{\mathrm {t}}}$ FSR scale	0.07
$\mathrm {t}\mathrm {W}$ FSR scale	0.02
$\mathrm {b}$ quark fragmentation	0.11
$\mathrm {b}$ hadron BF	0.07
Colour reconnection	0.17
DY background	0.24
$\mathrm {t}\mathrm {W}$ background	0.13
Diboson background	0.02
$\mathrm {W}$+jets background	0.04
${\mathrm {t}\overline{\mathrm {t}}}$ background	0.02
Statistical	0.14
MC statistical	0.36
Total $m_\mathrm {\mathrm {t}} ^{\mathrm {MC}}$ uncertainty	$^{+0.68}_{-0.73}$

## Extraction of $m_\mathrm {\mathrm {t}}$ and $\alpha _S (m_\mathrm {\mathrm {Z}})$ in the $\mathrm {\overline{MS}}$ scheme

The cross section value obtained in the simultaneous fit to $\sigma _{\mathrm {t}\overline{\mathrm {t}}}$ and $m_\mathrm {\mathrm {t}} ^{\mathrm {MC}}$ is used to extract $\alpha _S (m_\mathrm {\mathrm {Z}})$ and $m_\mathrm {\mathrm {t}}$ in the $\mathrm {\overline{MS}}$ renormalization scheme. For this purpose, the measured and the predicted cross sections are compared via a $\chi ^2$ minimization. The $\chi ^2$ fit is performed using the open-source QCD analysis framework xFitter  [76] and a $\chi ^2$ definition from Ref. [77]. The method to determine $m_\mathrm {\mathrm {t}}$ and $\alpha _S (m_\mathrm {\mathrm {Z}})$ is very similar to the one used in earlier CMS analyses to extract $\alpha _S (m_\mathrm {\mathrm {Z}})$ using jet cross section measurements, e.g. in Ref. [78].

It is assumed that the measured $\sigma _{\mathrm {t}\overline{\mathrm {t}}}$ is not affected by non-SM physics. The SM theoretical prediction for $\sigma _{\mathrm {t}\overline{\mathrm {t}}}$ at NNLO [6–9] is calculated using the Hathor  2.0 [79] program, interfaced with xFitter. This is the only available calculation to date that provides the $m_\mathrm {\mathrm {t}}$ definition in the $\mathrm {\overline{MS}}$ scheme. The top quark mass in the $\mathrm {\overline{MS}}$ scheme is denoted by $m_\mathrm {\mathrm {t}} (m_\mathrm {\mathrm {t}})$, following the convention of presenting the value of a running coupling at a fixed value. In the calculation, the renormalization and factorization scales, $\mu _\mathrm {r}$ and $\mu _\mathrm {f}$, are set to $m_\mathrm {\mathrm {t}} (m_\mathrm {\mathrm {t}})$. These are varied by a factor of two up and down, independently, avoiding cases where $\mu _\mathrm {f}$/$\mu _\mathrm {r}$ = 1/4 or 4, in order to estimate the uncertainty due to the missing higher-order corrections (referred to in the following as the scale variation uncertainty).

The values of $\alpha _S (m_\mathrm {\mathrm {Z}})$ and $m_\mathrm {\mathrm {t}}$ cannot be determined simultaneously, since both parameters alter the predicted $\sigma _{\mathrm {t}\overline{\mathrm {t}}}$ in such a way that any variation of one parameter can be compensated by a variation of the other. In the presented analysis, the values of $m_\mathrm {\mathrm {t}}$ and $\alpha _S (m_\mathrm {\mathrm {Z}})$ are therefore determined at fixed values of $\alpha _S (m_\mathrm {\mathrm {Z}})$ and $m_\mathrm {\mathrm {t}}$, respectively.

The four most recent PDF sets available [80] at NNLO are used: ABMP16nnlo [17], CT14nnlo [53], MMHT14nnlo [81], and NNPDF3.1nnlo [82]. While CT14nnlo does not use any ${\mathrm {t}\overline{\mathrm {t}}}$ data as input, the PDF sets ABMP16nnlo and MMHT14nnlo use measurements of inclusive ${\mathrm {t}\overline{\mathrm {t}}}$ cross sections at the Tevatron and LHC, and NNPDF3.1nnlo makes use of all available inclusive and differential ${\mathrm {t}\overline{\mathrm {t}}}$ cross section measurements. Using the currently available ${\mathrm {t}\overline{\mathrm {t}}}$ measurements has only a marginal effect on a global PDF and $\alpha _S (m_\mathrm {\mathrm {Z}})$ fit [17, 53]. The details of the PDFs relevant for this analysis are summarized in Table 4. In the MMHT14nnlo, CT14nnlo, and NNPDF3.1nnlo PDFs, the value of $\alpha _S (m_\mathrm {\mathrm {Z}})$ is assumed to be 0.118. In ABMP16nnlo, $\alpha _S (m_\mathrm {\mathrm {Z}})$ is fitted simultaneously with the PDFs. The ABMP16nnlo PDF employs the $\mathrm {\overline{MS}}$ scheme for the heavy-quark mass treatment in its determination. Similar to the value of $\alpha _S (m_\mathrm {\mathrm {Z}})$, the value of $m_\mathrm {\mathrm {t}} (m_\mathrm {\mathrm {t}})$ in the ABMP16nnlo set is obtained in a simultaneous fit with the PDFs. For the other PDFs, the values of $m_\mathrm {\mathrm {t}} ^{\text {pole}}$ are assumed, as listed in Table 4. Since the analysis is performed in the $\mathrm {\overline{MS}}$ scheme, the assumed $m_\mathrm {\mathrm {t}} ^{\text {pole}}$ of each PDF is converted into $m_\mathrm {\mathrm {t}} (m_\mathrm {\mathrm {t}})$ using the RunDec  [83, 84] code, according to the prescription by the corresponding PDF group.

**Table 4:** Values of the top quark pole mass $m_\mathrm {\mathrm {t}} ^{\text {pole}}$ and strong coupling constant $\alpha _S (m_\mathrm {\mathrm {Z}})$ used in the different PDF sets. Also shown are the corresponding $m_\mathrm {\mathrm {t}} (m_\mathrm {\mathrm {t}})$ values obtained using the RunDec  [83, 84] conversion, the number of loops in the conversion, and the $\alpha _S$ range used to estimate the PDF uncertainties

	ABMP16	NNPDF3.1	CT14	MMHT14
$m_\mathrm {\mathrm {t}} ^{\text {pole}}$ [$\text {Ge}\text {V}$]	170.37	172.5	173.3	174.2
RunDec loops	3	2	2	3
$m_\mathrm {\mathrm {t}} (m_\mathrm {\mathrm {t}})$ [$\text {Ge}\text {V}$]	160.86	162.56	163.30	163.47
$\alpha _S (m_\mathrm {\mathrm {Z}})$	0.116	0.118	0.118	0.118
$\alpha _S$ range	0.112−0.120	0.108−0.124	0.111−0.123	0.108−0.128

For each used PDF set, a series of $\alpha _S (m_\mathrm {\mathrm {Z}})$ values is provided. The PDF uncertainties for all sets correspond to a 68% confidence level ($\text {CL}$), whereby the uncertainties in the CT14nnlo PDF set are scaled down from 95% $\text {CL}$.

Because of the strong correlation between $\alpha _S$ and $m_\mathrm {\mathrm {t}}$ in the prediction of $\sigma _{\mathrm {t}\overline{\mathrm {t}}}$, for the $m_\mathrm {\mathrm {t}}$ extraction, the value of $\alpha _S (m_\mathrm {\mathrm {Z}})$ in the theoretical prediction is set to that of the particular PDF set. Similarly, in the theoretical prediction of $\sigma _{\mathrm {t}\overline{\mathrm {t}}}$ used for the $\alpha _S (m_\mathrm {\mathrm {Z}})$ determination, the value of $m_\mathrm {\mathrm {t}}$ is the one used in the PDF evaluation. The correlation of the values of $m_\mathrm {\mathrm {t}} (m_\mathrm {\mathrm {t}})$, $\alpha _S (m_\mathrm {\mathrm {Z}})$, and the proton PDFs in the prediction of $\sigma _{\mathrm {t}\overline{\mathrm {t}}}$ is also studied.

**Fig. 11:**
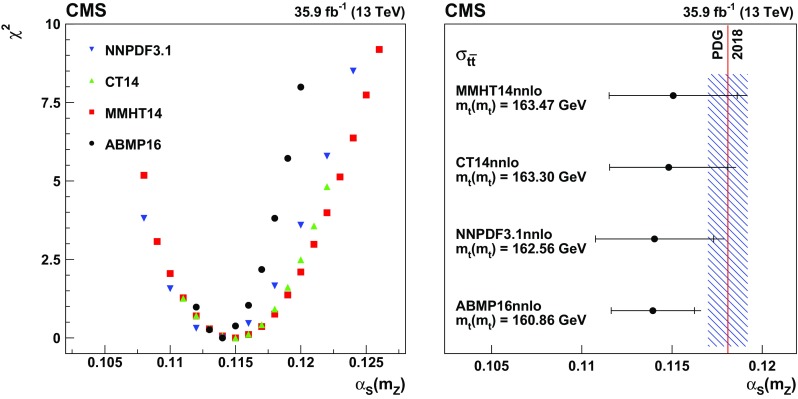
Left: $\chi ^2$ versus $\alpha _S$ obtained from the comparison of the measured $\sigma _{\mathrm {t}\overline{\mathrm {t}}}$ value to the NNLO prediction in the $\mathrm {\overline{MS}}$ scheme using different PDFs (symbols of different styles). Right: $\alpha _S (m_\mathrm {\mathrm {Z}})$ obtained from the comparison of the measured $\sigma _{\mathrm {t}\overline{\mathrm {t}}}$ value to the theoretical prediction using different PDF sets in the $\mathrm {\overline{MS}}$ scheme. The corresponding value of $m_\mathrm {\mathrm {t}} (m_\mathrm {\mathrm {t}})$ is given for each PDF set. The inner horizontal bars on the points represent the experimental and PDF uncertainties added in quadrature. The outer horizontal bars show the total uncertainties. The vertical line displays the world-average $\alpha _S (m_\mathrm {\mathrm {Z}})$ value [29], with the hatched band representing its uncertainty

To extract the value of $\alpha _S (m_\mathrm {\mathrm {Z}})$ from $\sigma _{\mathrm {t}\overline{\mathrm {t}}}$, the measured cross section is compared to the theoretical prediction, and for each $\alpha _S (m_\mathrm {\mathrm {Z}})$ member of each PDF set, the $\chi ^2$ is evaluated. In the case of ABMP16nnlo and NNPDF3.1nnlo, the complete set of PDF uncertainties is provided for each member of the $\alpha _S (m_\mathrm {\mathrm {Z}})$ series and is accounted for in the analysis. The uncertainties in the CT14nnlo and MMHT14nnlo PDFs are evaluated only for the central $\alpha _S (m_\mathrm {\mathrm {Z}})$ value of 0.118 and are used for each $\alpha _S (m_\mathrm {\mathrm {Z}})$ variant in the fit. The optimal value of $\alpha _S (m_\mathrm {\mathrm {Z}})$ is subsequently determined from a parabolic fit of the form

7 $$\begin{aligned} \chi ^2 (\alpha _S )=\chi ^2 _\text {min}+\left( \frac{\alpha _S - \alpha _S ^\text {min}}{\delta (\alpha _S ^\text {min})}\right) ^2 \end{aligned}$$

to the $\chi ^2 (\alpha _S )$ values. Here, $\chi ^2 _\text {min}$ is the $\chi ^2$ value at $\alpha _S = \alpha _S ^\text {min}$ and $\delta (\alpha _S ^\text {min})$ is the fitted experimental uncertainty in $\alpha _S ^\text {min}$, which also accounts for the PDF uncertainty. The $\chi ^2 (\alpha _S )$ scan is illustrated in Fig. 11 for the PDF sets used, demonstrating a clear parabolic behaviour. To estimate the scale variation uncertainties, this procedure is repeated with $\mu _\mathrm {r}$ and $\mu _\mathrm {f}$ being varied, and the largest deviations of the resulting values of $\alpha _S ^\text {min}$ from that of the central scale choice are considered as the corresponding uncertainties. The values of the $\alpha _S (m_\mathrm {\mathrm {Z}})$ obtained using different PDFs are listed in Table 5 and shown in Fig. 11. The uncertainties in the measured $\sigma _{\mathrm {t}\overline{\mathrm {t}}}$ and the PDF contribute about equally to the resulting $\alpha _S (m_\mathrm {\mathrm {Z}})$ uncertainty.

**Table 5:** Values of $\alpha _S (m_\mathrm {\mathrm {Z}})$ with their uncertainties obtained from a comparison of the measured $\sigma _{\mathrm {t}\overline{\mathrm {t}}}$ value to the NNLO prediction in the $\mathrm {\overline{MS}}$ scheme using different PDF sets. The first uncertainty is the combination of the experimental and PDF uncertainties, and the second is from the variation of the renormalization and factorization scales

PDF set	$\alpha _S (m_\mathrm {\mathrm {Z}})$
ABMP16	0.1139 ± 0.0023 (fit + PDF) $^{+0.0014}_{-0.0001}$ (scale)
NNPDF3.1	0.1140 ± 0.0033 (fit + PDF) $^{+0.0021}_{-0.0002}$ (scale)
CT14	0.1148 ± 0.0032 (fit + PDF) $^{+0.0018}_{-0.0002}$ (scale)
MMHT14	0.1151 ± 0.0035 (fit + PDF) $^{+0.0020}_{-0.0002}$ (scale)

The values of $\alpha _S (m_\mathrm {\mathrm {Z}})$ obtained using different PDF sets are consistent among each other and are in agreement with the world-average value [29] within the uncertainties, although suggesting a smaller value of $\alpha _S (m_\mathrm {\mathrm {Z}})$. The value of $\alpha _S (m_\mathrm {\mathrm {Z}})$ is also in good agreement with the recent result of the analysis in Ref. [85] of jet production in deep-inelastic scattering using the NNLO calculation by the H1 experiment, and is of comparable precision.

The same procedure is used to extract $m_\mathrm {\mathrm {t}} (m_\mathrm {\mathrm {t}})$ by fixing $\alpha _S (m_\mathrm {\mathrm {Z}})$ to the nominal value at which the used PDF is evaluated. The fit is performed by varying $m_\mathrm {\mathrm {t}} (m_\mathrm {\mathrm {t}})$ in a 5-$\text {Ge}\text {V}$ range around the central value used in each PDF. The uncertainties related to the variation of $\alpha _S (m_\mathrm {\mathrm {Z}})$ in the PDF are estimated by repeating the fit using the PDF eigenvectors with $\alpha _S (m_\mathrm {\mathrm {Z}})$ varied within its uncertainty, as provided by NNPDF3.1nnlo, MMHT2014nnlo, and CT14nnlo. In the case of ABMP16nnlo, the value of $\alpha _S (m_\mathrm {\mathrm {Z}})$ is a free parameter in the PDF fit and its uncertainty is implicitly included in the ABMP16nnlo PDF uncertainty eigenvectors. The resulting $m_\mathrm {\mathrm {t}} (m_\mathrm {\mathrm {t}})$ values are summarized in Table 6, where the fit uncertainty corresponds to the precision of the $\sigma _{\mathrm {t}\overline{\mathrm {t}}}$ measurement. The results obtained with different PDF sets are in agreement, although the ABMP16nnlo PDF set yields a systematically lower value. This difference is expected and has its origin in a larger value of $\alpha _S (m_\mathrm {\mathrm {Z}}) = 0.118$ assumed in the NNPDF3.1, MMHT2014, and CT14 PDFs.

**Table 6:** Values of $m_\mathrm {\mathrm {t}} (m_\mathrm {\mathrm {t}})$ obtained from the comparison of the $\sigma _{\mathrm {t}\overline{\mathrm {t}}}$ measurement with the NNLO predictions using different PDF sets. The first uncertainty shown comes from the experimental, PDF, and $\alpha _S (m_\mathrm {\mathrm {Z}})$ uncertainties, and the second from the variation in the renormalization and factorization scales

PDF set	$m_\mathrm {\mathrm {t}} (m_\mathrm {\mathrm {t}})$ ($\text {Ge}\text {V}$)
ABMP16	161.6 ± 1.6 (fit + PDF + $\alpha _S$) $^{+0.1}_{-1.0}$ (scale)
NNPDF3.1	164.5 ± 1.6 (fit + PDF + $\alpha _S$) $^{+0.1}_{-1.0}$ (scale)
CT14	165.0 ± 1.8 (fit + PDF + $\alpha _S$) $^{+0.1}_{-1.0}$ (scale)
MMHT14	164.9 ± 1.8 (fit + PDF + $\alpha _S$) $^{+0.1}_{-1.1}$ (scale)

**Fig. 12:**
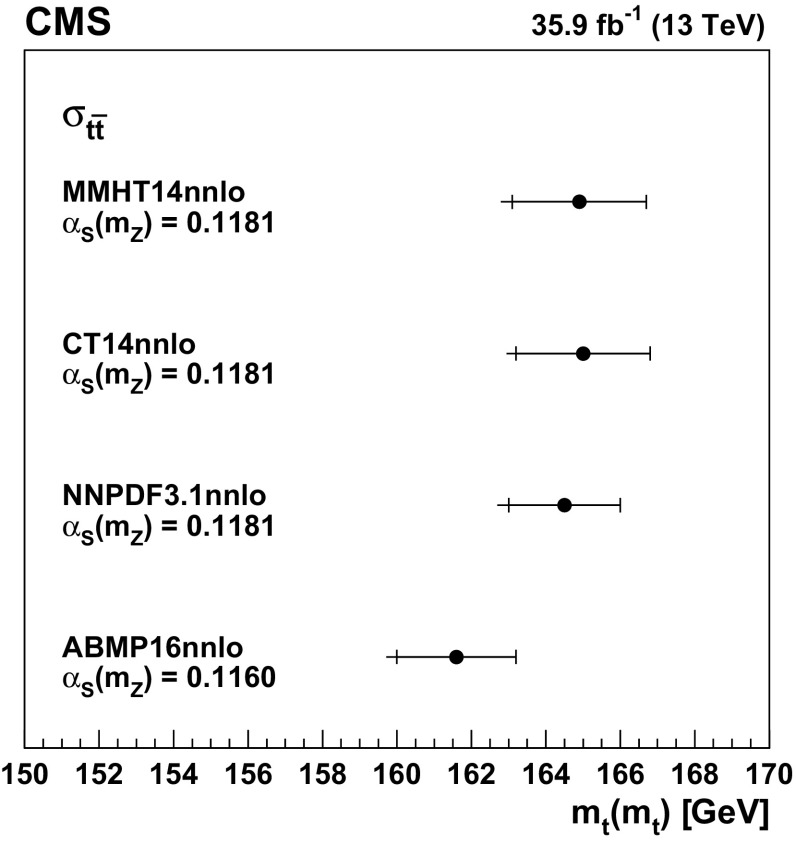
Values of $m_\mathrm {\mathrm {t}} (m_\mathrm {\mathrm {t}})$ obtained from comparing the $\sigma _{\mathrm {t}\overline{\mathrm {t}}}$ measurement to the theoretical NNLO predictions using different PDF sets. The inner horizontal bars on the points represent the quadratic sum of the experimental, PDF, and $\alpha _S (m_\mathrm {\mathrm {Z}})$ uncertainties, while the outer horizontal bars give the total uncertainties

The values of $m_\mathrm {\mathrm {t}} (m_\mathrm {\mathrm {t}})$ are in agreement with those originally used in the evaluation of each PDF set. The results are shown in Fig. 12 for the four different PDFs used.

**Fig. 13:**
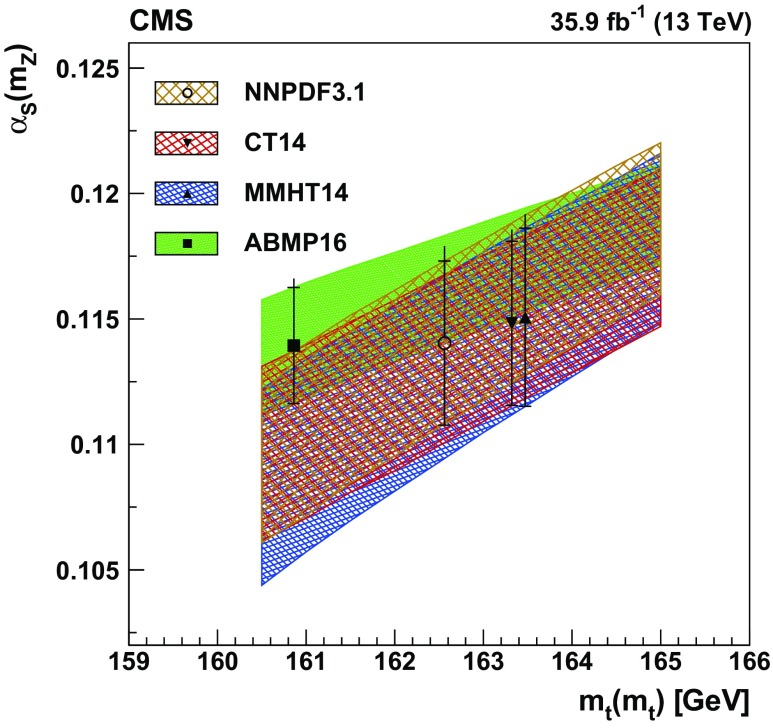
Values of $\alpha _S (m_\mathrm {\mathrm {Z}})$ obtained in the comparison of the $\sigma _{\mathrm {t}\overline{\mathrm {t}}}$ measurement to the NNLO prediction using different PDFs, as a function of the $m_\mathrm {\mathrm {t}} (m_\mathrm {\mathrm {t}})$ value used in the theoretical calculation. The results from using the different PDFs are shown by the bands with different shadings, with the band width corresponding to the quadratic sum of the experimental and PDF uncertainties in $\alpha _S (m_\mathrm {\mathrm {Z}})$. The resulting measured values of $\alpha _S (m_\mathrm {\mathrm {Z}})$ are shown by the different style points at the $m_\mathrm {\mathrm {t}} (m_\mathrm {\mathrm {t}})$ values used for each PDF. The inner vertical bars on the points represent the quadratic sum of the experimental and PDF uncertainties in $\alpha _S (m_\mathrm {\mathrm {Z}})$, while the outer vertical bars show the total uncertainties

The dependence of the $\alpha _S (m_\mathrm {\mathrm {Z}})$ result on the assumption on $m_\mathrm {\mathrm {t}} (m_\mathrm {\mathrm {t}})$ is investigated for each PDF by performing the $\chi ^2 (\alpha _S )$ scan for ten values of $m_\mathrm {\mathrm {t}} (m_\mathrm {\mathrm {t}})$ varying from 160.5 to 165.0$\,\text {Ge}\text {V}$. A linear dependence is observed, as shown in Fig. 13.

## Extraction of $m_\mathrm {\mathrm {t}}$ in the pole mass scheme

The extraction of $m_\mathrm {\mathrm {t}}$ is repeated in the pole mass scheme using the Top++  2.0 program [52], which employs the calculation of $\sigma _{\mathrm {t}\overline{\mathrm {t}}}$ at NNLO, improved by the NNLL soft-gluon resummation. The results are summarized in Table 7. The scale variation uncertainties are estimated in the same way as in the case of the $m_\mathrm {\mathrm {t}} (m_\mathrm {\mathrm {t}})$ extraction. These uncertainties are larger than those determined in the $\mathrm {\overline{MS}}$ scheme. This is because of the better convergence of the perturbative series when using the $\mathrm {\overline{MS}}$ renormalization scheme in the calculation of $\sigma _{\mathrm {t}\overline{\mathrm {t}}}$.

**Table 7:** Values of $m_\mathrm {\mathrm {t}} ^{\text {pole}}$ obtained by comparing the $\sigma _{\mathrm {t}\overline{\mathrm {t}}}$ measurement with predictions at NNLO+NNLL using different PDF sets

PDF set	$m_\mathrm {\mathrm {t}} ^{\text {pole}}$ ($\text {Ge}\text {V}$)
ABMP16	169.9 ± 1.8 (fit + PDF + $\alpha _S$) $^{+0.8}_{-1.2}$ (scale)
NNPDF3.1	173.2 ± 1.9 (fit + PDF + $\alpha _S$) $^{+0.9}_{-1.3}$ (scale)
CT14	173.7 ± 2.0 (fit + PDF + $\alpha _S$) $^{+0.9}_{-1.4}$ (scale)
MMHT14	173.6 ± 1.9 (fit + PDF + $\alpha _S$) $^{+0.9}_{-1.4}$ (scale)

## Summary

A measurement of the top quark–antiquark pair production cross section $\sigma _{\mathrm {t}\overline{\mathrm {t}}}$ by the CMS Collaboration in proton–proton collisions at a centre-of-mass energy of 13$\,\text {Te}\text {V}$ is presented, corresponding to an integrated luminosity of $35.9{\,\text {fb}^{-1}}$. Assuming a top quark mass in the simulation of $m_\mathrm {\mathrm {t}} ^{\mathrm {MC}} = 172.5 \,\text {Ge}\text {V}$, a visible cross section is measured in the fiducial region using dilepton events ($\mathrm {e}$ $^{\pm }$ $\mathrm {\mu }$ $^{{\mp }}$, $\mathrm {\mu ^+}\mathrm {\mu ^-}$, $\mathrm {e}^+\mathrm {e}^-$) and then extrapolated to the full phase space. The total ${\mathrm {t}\overline{\mathrm {t}}}$ production cross section is found to be $\sigma _{\mathrm {t}\overline{\mathrm {t}}} = 803 \pm 2 \,\text {(stat)} \pm 25 \,\text {(syst)} \pm 20 \,\text {(lumi)} \,\text {pb}$. The measurement is in good agreement with the theoretical prediction calculated to next-to-next-to-leading order in perturbative QCD, including soft-gluon resummation to next-to-next-to-leading logarithm.

The measurement is repeated including the top quark mass in the powheg simulation as an additional free parameter in the fit. The sensitivity to $m_\mathrm {\mathrm {t}} ^{\mathrm {MC}}$ is maximized by fitting the minimum invariant mass found when combining the charged leptons with the $\mathrm {b}$ jets in an event. This yields a cross section of $\sigma _{\mathrm {t}\overline{\mathrm {t}}} = 815 \pm 2 \,\text {(stat)} \pm 29 \,\text {(syst)} \pm 20 \,\text {(lumi)} \,\text {pb}$ and a value of $m_\mathrm {\mathrm {t}} ^{\mathrm {MC}} = 172.33 \pm 0.14 \,\text {(stat)} \,^{+0.66}_{-0.72} \,\text {(syst)} \,\text {Ge}\text {V}$, in good agreement with previous measurements. The value of $\sigma _{\mathrm {t}\overline{\mathrm {t}}}$ obtained in the simultaneous fit is further used to extract the values of the top quark mass and the strong coupling constant at next-to-next-to-leading order in the minimal subtraction renormalization scheme, as well as the value of the top quark pole mass for different sets of parton distribution functions.

## Data Availability

This manuscript has no associated data or the data will not be deposited . [Authors’ comment: Release and preservation of data used by the CMS Collaboration as the basis for publications is guided by the CMS policy as written in its document “CMS data preservation, re-use and open access policy” (https://cms-docdb.cern.ch/cgi-bin/PublicDocDB/RetrieveFile?docid=6032&filename=CMSDataPolicyV1.2.pdf&version=2).]
